# Ecological principle meets cancer treatment: treating children with acute myeloid leukemia with low-dose chemotherapy

**DOI:** 10.1093/nsr/nwz006

**Published:** 2019-01-22

**Authors:** Yixin Hu, Aili Chen, Xinchang Zheng, Jun Lu, Hailong He, Jin Yang, Ya Zhang, Pinpin Sui, Jingyi Yang, Fuhong He, Yi Wang, Peifang Xiao, Xin Liu, Yinmei Zhou, Deqing Pei, Cheng Cheng, Raul C Ribeiro, Shaoyan Hu, Qian-fei Wang

**Affiliations:** 1 Department of Hematology and Oncology, Children's Hospital of Soochow University, Suzhou 215025, China; 2 CAS Key Laboratory of Genomic and Precision Medicine, Beijing Institute of Genomics, Chinese Academy of Sciences, Beijing 100101, China; 3 University of Chinese Academy of Sciences, Beijing 100049, China; 4 Department of Pediatrics, Nothern Jiangsu People's Hospital, Yangzhou 225001, China; 5 Department of Biostatistics, St. Jude Children's Research Hospital, Memphis TN 38105, USA; 6 Department of Oncology and Global Medicine, International Outreach Program, St. Jude Children's Research Hospital, Memphis, TN 38105, USA

**Keywords:** low-dose chemotherapy, acute myeloid leukemia, G-CSF

## Abstract

Standard chemotherapy regimens for remission induction of pediatric acute myeloid leukemia (AML) are associated with significant morbidity and mortality. We performed a cohort study to determine the impact of reducing the intensity of remission induction chemotherapy on the outcomes of selected children with AML treated with a low-dose induction regimen plus granulocyte colony stimulating factor (G-CSF) (low-dose chemotherapy (LDC)/G-CSF). Complete response (CR) after two induction courses was attained in 87.0% (40/46) of patients receiving LDC/G-CSF. Post-remission therapy was offered to all patients, and included standard consolidation and/or stem cell transplantation. During the study period, an additional 94 consecutive children with AML treated with standard chemotherapy (SDC) for induction (80/94 (85.1%) of the patients attained CR after induction II, *P* = 0.953) and post-remission. In this non-randomized study, there were no significant differences in 4-year event-free (67.4 vs. 70.7%; *P* = 0.99) and overall (70.3 vs. 74.6%, *P* = 0.69) survival in the LDC/G-CSF and SDC cohorts, respectively. After the first course of induction, recovery of white blood cell (WBC) and platelet counts were significantly faster in patients receiving LDC/G-CSF than in those receiving SDC (11.5 vs. 18.5 d for WBCs (*P* < 0.001); 15.5 vs. 22.0 d for platelets (*P* < 0.001)). To examine the quality of molecular response, targeted deep sequencing was performed. Of 137 mutations detected at diagnosis in 20 children who attained hematological CR after two courses of LDC/G-CSF (*n* = 9) or SDC (*n* = 11), all of the mutations were below the reference value (variant allelic frequency <2.5%) after two courses, irrespective of the treatment group. In conclusion, children with AML receiving LDC/G-CSF appear to have similar outcomes and mutation clearance levels, but significantly lower toxicity than those receiving SDC. Thus, LDC/G-CSF should be further evaluated as an effective alternative to remission induction in pediatric AML.

## INTRODUCTION

Malignant tumors, including acute myeloid leukemia (AML), are widely considered to have a genetically heterogeneous cell population [1,2]. Recent studies have found that the intratumor genetic diversity is much higher than previously expected [3,4]. For example, the number of coding mutations in a single hepatocarcinoma was estimated to be greater than 100 million under a neutrality model [3]. In this model, the high intratumor mutation load would be a consequence of neutral (non-Darwinian) molecular evolution whereby mutations are not eliminated by natural selection (Darwinian). Moreover, population genetic analyses support the neutral model during tumor growth [5,6]. Except for the strong positive selection of a few driving mutations, most of the tumor cells are subject to little natural selection in the absence of therapeutic intervention and changes in the microenvironment. However, an originally neutral mutation could later become adaptive in a changed environment, so called the ‘Dykhuizen–Hartl effect’ [7]. Therefore, new mutations are constantly occurring during tumor formation, which likely gives rise to drug-resistant mutations at a very low frequency.

With the pre-existence of small drug-resistant subclones, the elimination of a dominant drug-sensitive clone by current chemotherapy, usually given at the maximum tolerated dose, might allow the competitive release of resistant subclones to undergo accelerated growth in a resource-rich environment, resulting in more rapid disease progression or relapse [8,9]. These observations are consistent with the evolutionary ecological theory of biological control, in which successful eradication in exotic species using high-dose pesticides may be impossible, and usually results in the rapid emergence of resistant strains [10]. Hence, reducing chemotherapy drug dosage may be a logical way to maintain a stable population of sensitive subclones, thereby restricting the growth of resistant cells. Gatenby and colleagues have devised adaptive therapy, which requires progressively lower dosing or even omitted schedules, to maximize time to progression by stabilizing tumor size in animal models [11,12]. Such a low-dose adaptive concept may provide a novel strategy for treating non-curable advanced or metastatic tumors; however, it is not clear whether reduced chemotherapy intensity can be clinically effective when cure is the goal.

Pediatric AML is generally managed with two courses of near-myeloablative therapy to induce remission, followed by two-to-three additional courses of chemotherapy or hematopoietic cell transplantation (HCT) in selected patients for post-remission therapy [13,14]. However, intensive induction therapy is associated with life-threatening complications, as patients are most vulnerable to the effects of high leukemia burden and profound marrow suppression [15]. Treatment-related deaths are particularly high in places with limited resources [16].

A low-dose chemotherapy (LDC) regimen featuring one-tenth of the standard dose of chemotherapeutic drug cytarabine and one-half of the dose of anthracycline in conjunction with granulocyte colony stimulating factor (G-CSF), was used in induction and consolidation therapies for elderly patients with AML who were medically unfit to receive intensive chemotherapy [17]. Approximately 35–40% of patients given LDC/G-CSF attained complete response (CR) [18]. However, this strategy was originally aimed at improving the quality of life but not curing the leukemia. Whether LDC/G-CSF regimens followed by standard post-remission approaches can eradicate AML remains unknown. Further, the efficacy of LDC/G-CSF has not been tested in children with *de novo* AML.

Given the efficacy and good tolerance of LDC/G-CSF regimens in older patients, we report here a cohort study of children with newly diagnosed AML treated with the LDC/G-CSF regimen. The clinical and biological features, and outcomes of children with AML receiving at least one induction cycle of LDC/G-CSF were compare with those receiving standard-dose chemotherapy (SDC), to investigate whether LDC could achieve similar clinical efficacy as SDC.

## RESULTS

### Patient characteristics

Between July 2012 and May 2018, 140 children (<15 years old) with *de novo* AML were treated at our hospital. For induction I, 46 received LDC/G-CSF and 94 received SDC. Median age of 82 males and 58 females was 81 months (range, 5–170 months). Median white blood cell (WBC) count was 16.5 × 10^9^/L (range, 0.4–606.0 × 10^9^/L). According to World Health Organization (WHO) criteria, 85 patients had AML with recurrent cytogenetic abnormalities and 55 had AML that was not otherwise specified (Table 1).

**Table 1.** Baseline features and treatment responses of 140 children with AML.

Features	LDC/G-CSF *N* (%)	SDC *N* (%)	Total *N* (%)	*P* value
Gender, *n* (%)	46	94	140	0.719^a^
Female	18 (39.1)	40 (42.6)	58 (41.4)	
Male	28 (60.9)	54 (57.4)	82 (58.6)	
Age (months)	46	94	140	0.014^b^
Median	54	93	81	
Range	10–149	5–170	5–170	
WBC count (×10^9^/L)	46	94	140	
Median	9.97	22.06	16.47	0.002^b^
Range	1.33–69.51	0.42–606.00	0.42–606.00	
Gene rearrangements, *n* (%)	46	94	140	0.206^a^
*RUNX1-RUNX1T1*	20 (43.5)	34 (36.2)	54 (38.6)	
*MLL*r	5 (10.9)	16 (17.0)	21 (15.0)	
*CBF/MYH11*	2 (4.3)	14 (14.9)	16 (11.4)	
*BCR-ABL*	0 (0.0)	1 (1.0)	1 (0.7)	
Negative	19 (41.3)	29 (30.9)	48 (34.3)	
Gene mutations, *n* (%)^c^	46	86	132	0.174^a^
*KIT*	10 (21.7)	22 (25.6)	32 (24.2)	
*NPM1*	1 (2.2)	1 (1.2)	2 (1.5)	
*FLT3*-ITD	0 (0)	11 (12.8)	11 (8.3)	
*CEBPα* double mutation	3 (6.5)	5 (5.8)	8 (6.1)	
*PTPN11*	2 (4.3)	2 (2.3)	4 (3.1)	
Negative	30 (65.2)	45 (52.3)	75 (56.8)	
Cytogenetics, *n* (%)	46	94	140	0.466^a^
Favorable	21 (45.6)	41 (43.6)	62 (44.3)	
Intermediate	17 (37.0)	39 (41.5)	56 (40.0)	
Adverse	7 (15.2)	8 (8.5)	15 (10.7)	
Undetectable	1 (2.2)	6 (6.4)	7 (5.0)	
AML risk group, *n* (%)	46	94	140	0.219^a^
Low risk	13 (28.3)	31 (33.0)	44 (31.4)	
Intermediate risk	25 (54.3)	37 (39.4)	62 (44.3)	
High risk	8 (17.4)	26 (27.6)	34 (24.3)	
First induction, *n* (%)	46	94	140	0.570^a^
CR	34 (73.9)	64 (68.1)	98 (70.0)	
PR	5 (10.9)	17 (18.1)	22 (15.7)	
NR	7 (15.2)	13 (13.8)	20 (14.3)	
Second induction, *n* (%)	44^e^	90	134	0.953^a^
CR	40 (90.9)	80 (88.9)	120 (89.6)	
PR	1 (2.3)	4 (4.4)	5 (3.7)	
NR	2 (4.5)	5 (5.6)	7 (5.2)	
Unknown	1 (2.3)	1 (1.1)	2 (1.5)	
HCT, *n* (%)	46	94	140	0.820^a^
No	25 (54.3)	53 (56.4)	78 (55.7)	
Yes	21 (45.7)	41 (43.6)	62 (44.3)	
Relapse, *n* (%)	46	94	140	0.514^a^
No	41 (89.1)	80 (85.1)	121 (86.4)	
Yes	5 (10.9)	14 (14.9)	19 (13.6)	
Deaths, *n* (%)	46	94	140	0.835^a^
No	34 (73.9)	71 (75.5)	105 (75.0)	
Yes	12 (26.1)	23 (24.5)	35 (25.0)	
Toxic deaths, *n* (%)	46	94	140	0.719^a^^,^^d^
No	42 (91.3)	84 (84.4)	126 (90.0)	
Yes	4 (8.7)	10 (10.6)	14 (10.0)	
Induction I	0 (0.0)	0 (0.0)	0 (0.0)	
Induction II	0 (0.0)	2 (2.1)	2 (1.4)	
Post-induction	1 (2.2)	4 (4.3)	5 (3.6)	
Post-HCT	3 (6.5)	4 (4.3)	7 (5.0)	
Abandonment, *n* (%)	46	94	140	0.754^a^^,^^d^
No	43 (95.3)	86 (91.5)	129 (92.1)	
Yes	3 (6.5)	8 (8.5)	11 (7.9)	

AML: acute myeloid leukemia; ANC: absolute neutrophil count; LDC/G-CSF: low-dose chemotherapy concurrent with G-CSF; SDC: standard-dose chemotherapy; WBC: white blood cell; CR: complete response; HCT: hematopoietic cell transplant; NR: no response; PR: partial response.

^a^Exact Pearson's chi-squared test.

^b^Wilcoxon rank-sum test.

^c^Eight patients did not receive gene mutation test.

^d^ *P* value is given for comparing the first 2 rows (No and Yes) between LDC/G-CSF and SDC. Subsequent rows show the distribution of Yes patients by treatment phase.

^e^Sixteen patients received SDC for induction II.

Patients treated with LDC/G-CSF were significantly younger (median, 54 months (range, 10–149 months) vs. 93 months (range, 5–170 months); *P* = 0.014) and had lower initial WBC counts (median, 10.0 × 10^9^/L (range, 1.3–69.5 × 10^9^/L) vs. 22.1 × 10^9^/L (0.4–606.0 × 10^9^/L); *P* = 0.002) than those treated with SDC. There were no significant differences between other presenting clinical or biological features between both groups (*P* = 0.466; Table 1).

### Treatment outcome and response

#### Induction remission in patients assigned to the LDC/G-CSF group

Thirty-four of 46 (73.9%) children who received the first course of LDC/G-CSF (induction I) attained remission. Failure to attain remission was observed in 12 (26.1%) patients because of no response (NR) in 7 and partial response (PR) in five patients (Table 1 and Supplementary Fig. S1). A second course of LDC/G-CSF (induction II) was administered to 28 of the original 46 patients. Two patients abandoned treatment and 16 patients (nine cases with CR, two cases with PR and five cases with NR) received SDC for induction II.

Overall, after inductions I and II, 40/46 (87.0%) of patients originally assigned to the LDC/G-CSF group attained remission. Persistent disease was the most common cause of failure in achieving CR after induction II (Table 1 and Supplementary Fig. S1). No toxic deaths were observed in this group.

#### Induction remission in patients assigned to the SDC group

Sixty-four of 94 children assigned to the SDC group (68.1%) attained remission after induction I. Failure to attain remission was observed in 30 patients (NR in 13 and PR in 17 patients). After induction II, 80/94 (85.1%) of the patients attained remission. Causes of failure included persistent disease (12 cases) and treatment-related death (two cases) (Table 1 and Supplementary Fig. S1).

#### Post-remission therapy

Of 134 patients completing both inductions, 128 received post-induction therapy (Table 1 and Supplementary Fig. S1). Of the remaining six patients, four died from leukemia and two from toxicity. Of 128 patients receiving post-induction therapy, one from each group abandoned treatment.

**Figure 1.**
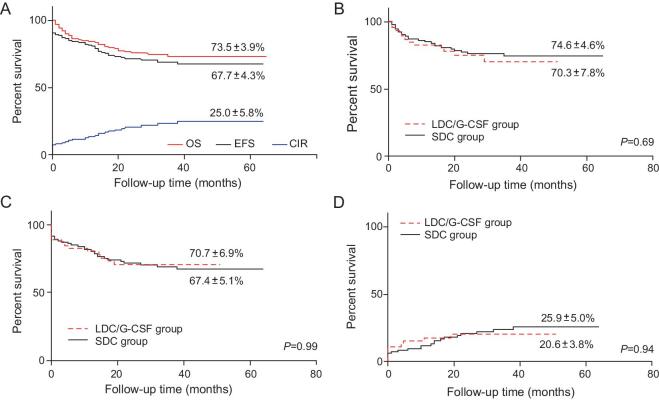
Survival and cumulative incidence of relapse in 140 children with acute myeloid leukemia. A. Overall survival (OS), event-free survival (EFS) and cumulative incidence of relapse (CIR). B. OS according to regimen. C. EFS according to regimen. D. CIR according to regimen.

By 31 May 2018, 105 patients, including 34/46 (73.9%) and 71/94 (75.5%) initially in the LDC/G-CSF and SDC groups, respectively, were alive without evidence of disease. At a median follow-up of 2.5 years (range, 0.08–5.41 years), 118 (84.3%), 80 (57.1%), 56 (40.0%) and 30 (21.4%) patients were followed for at least 1, 2, 3 or 4 years, respectively. The 4-year OS and event-free survival (EFS) for 140 children with AML were 73.5 ± 3.9% and 67.7 ± 4.3%, respectively (Fig. 1A), and 4-year cumulative incidence of relapse (CIR) was 25.0 ± 5.8% (Fig. 1A).

There were no significant differences in OS and EFS between the LDC/G-CSF and SDC groups (*P* = 0.69 and *P* = 0.99, respectively, log-rank test stratified by AML risk; Fig. 1B and C). For the LDC/G-CSF and SDC groups, 4-year OS was 70.3 ± 7.8 vs. 74.6 ± 4.6% and EFS was 67.4 ± 5.1% vs. 70.7 ± 6.9%, respectively.

Multivariate Cox regression with chemotherapy dose groups (LDC/G-CSF and SDC) and the AML risk group (low, intermediate and high) as covariates (Supplementary Table S1) showed no significant association of chemotherapy dose with OS (hazard ratio (HR) = 0.955, *P* = 0.91) or EFS (HR = 1.052; *P* = 0.885). High-risk AML was significantly associated with OS (HR = 4.04, *P* = 0.009) and EFS (HR = 4.58, *P* = 0.0015). Even when gender, age, initial WBC and HCT were added as covariates, chemotherapy dose was not significantly associated with OS or EFS (Supplementary Table S2).

Similarly, 4-year CIR rates in the LDC/G-CSF and SDC groups were not significantly different (20.6 ± 3.8% vs. 25.9 ± 5.0%, respectively (risk-stratified Gray's test, *P* = 0.94; Fig. 1D)). Multivariate Fine–Gray regression analyses with chemotherapy dose group, AML risk classification, gender, age, initial WBC and HCT as covariates revealed that chemotherapy dose did not affect relapse risk (HR = 0.74, *P* = 0.49; Supplementary Table S2).

Because only patients with WBC count <70 × 10^9^/L received LDC/G-CSF, we compared OS and EFS of the LDC/G-CSF group and a SDC subgroup with WBC count <70 × 10^9^/L. OS and EFS were not significantly different between both groups (log-rank test *P* = 0.27 and *P* = 0.67, respectively; Supplementary Fig. S2A and B). In this subset, multivariate Cox regression with chemotherapy dose group, risk, gender, age, initial WBC and HCT as covariates revealed no effect of chemotherapy dose on OS (HR = 0.57, *P = *0.20). AML (HR = 4.02, *P* = 0.038) and bone marrow (BM) transplant (BMT; HR = 8.81; *P* = 0.0039) were significant factors for death (Supplementary Table S3). Multivariate Cox regression analysis also showed no effect of chemotherapy dose on EFS (HR = 0.75, *P* = 0.46). High-risk AML (HR = 4.26, *P* = 0.0119) was significantly associated with EFS. Similar results were obtained for risk of relapse (Supplementary Table S3).

### Toxicity

**Figure 2.**
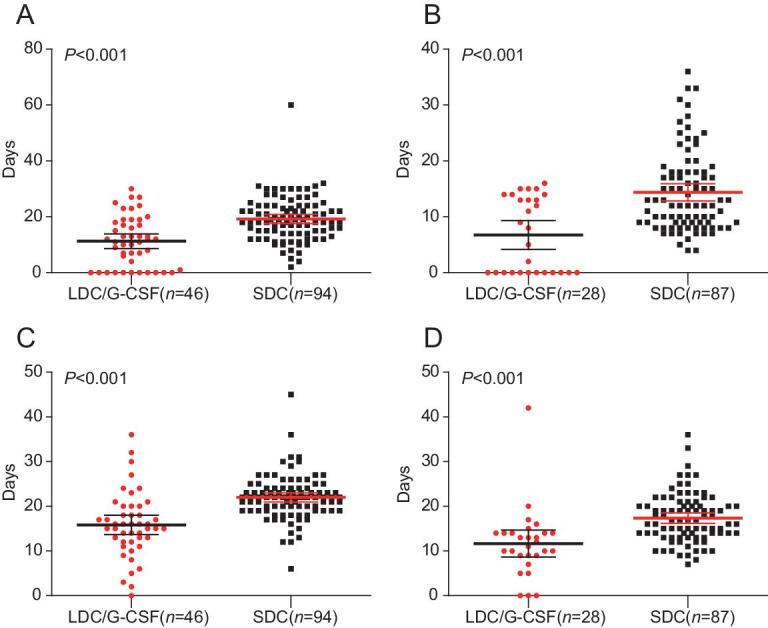
Duration (median number of days) of neutropenia and thrombocytopenia during induction chemotherapy. A. Number of days to reach neutrophil count of at least 0.5 × 10^9^/L after first induction according to treatment group. B. Number of days to reach neutrophil count of at least 0.5 × 10^9^/L after second induction according to treatment group. C. Number of days to reach platelet count of at least 20.0 × 10^9^/L after first induction. D. Number of days to reach platelet count of at least 20.0 × 10^9^/L after second induction.

During inductions I and II, two patients receiving SDC died of toxicity. The most common toxicity was myelosuppression. The median number of days to reach an absolute neutrophil count (ANC) of 0.5 × 10^9^/L was significantly shorter for those in the LDC/G-CSF than the SDC group after both inductions I (11.5 vs. 18.5 d, *P* < 0.001; Supplementary Table S4 and Fig. 2A) and II (6.5 vs. 12.0 d, *P* < 0.001; Supplementary Table S4 and Fig. 2B). Strikingly, in 11/46 patients in the LDC/G-CSF group attaining at least PR after induction I, ANCs never dropped below 0.5 × 10^9^/L. The median number of days to reach platelet counts of 20.0 × 10^9^/L was also significantly shorter for the LDC/G-CSF group than the SDC group after inductions I (15.5 vs. 22.0 d, *P* < 0.001; Supplementary Table S4 and Fig. 2C) and II (11.5 vs. 17.0 d, *P* < 0.001; Supplementary Table S4, Fig. 2D).

Grade III or IV infectious complications occurred in 4 (8.7%) and 23 (24.4%) patients receiving LDC/G-CSF and SDC regimens, respectively (*P* < 0.001). Pulmonary infection and sepsis were the most common complications. Non-hematologic toxicities were mild (mostly grade I or II) in both groups.

### Molecular remission after induction chemotherapy and during follow-up

Because of the possibility of residual leukemia-associated genetic variants persisting after induction treatment with LDC/G-CSF, we investigated the quality and duration of molecular remission by targeted deep sequencing performed in 86 samples from 20 patients who attained morphologic remission after induction I with the LDC/G-CSF (*n* = 9) or SDC (*n* = 11) regimens. There were no significant differences in clinical and cytogenetic characteristics by treatment group (Supplementary Table S5).

Median follow-up sampling time from diagnosis was 9 months (range 1–25 months). A total of 149 mutations identified by exome sequencing, including 137 found in diagnosis samples and 12 identified in relapsed samples, were analyzed (Whole exome sequencing data not shown), and mean coverage was 14 010.99 × (range, 206–120 429). At diagnosis, the average number of mutations per patient for the LDC/G-CSF and SDC groups was 7.6 (range 4–13) and 6.3 (range 2–13), respectively. The average variant allelic frequency (VAF) at diagnosis was 29.4 and 35.3% for the LDC/G-CSF and SDC groups, respectively (68 and 69, respectively, for the LDC/G-CSF or SDC groups; 2–13 mutations per patient; mean, 6.85).

**Figure 3.**
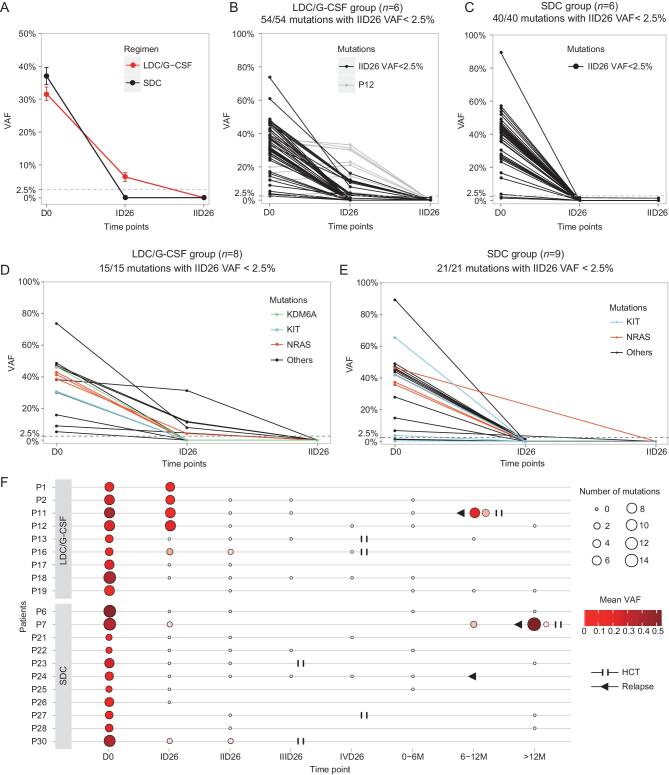
Clearance patterns of mutations detected by sequencing of acute myeloid leukemia patients according to treatment regimen. A. Average reduction in the allelic frequency of all mutations (*n* = 137) according to treatment intensity after first (ID_26_) and second induction (IID_26_) in 20 patients (P) in morphologic remission after first remission. B. Clearance patterns of mutations (*n* = 54) detected at diagnosis (D_0_), and after the ID_26_ and IID_26_ induction courses in six patients treated with the low-dose chemotherapy plus G-CSF (LDC/G-CSF) regimen. Persistent mutations (light gray) were seen in a single patient (P12). C. Clearance patterns of mutations (*n* = 40) detected at diagnosis (D_0_), and after the ID_26_ and IID_26_ induction courses of six patients treated with the standard-dose chemotherapy (SDC) regimen. D. Clearance patterns of 15 leukemia-associated RMGs detected at D_0_, and after the ID_26_) and IID_26_ induction courses, in eight patients treated with the LDC/G-CSF regimen, including one KDM6A mutation, two KIT mutations and three NRAS mutations. E. Clearance patterns of 21 leukemia-associated RMGs detected at D_0_, and after the ID_26_ and IID_26_ induction courses of nine patients treated with the SDC regimen, including five KIT mutations and four NRAS mutations. F. Clearance of mutations and average reduction of their variant allelic frequency (VAF) detected by deep sequencing at different time points during and after completion of treatment.

A reference value of VAF < 2.5% has been defined as molecular remission based on a previous report [19]. After inductions I and II, there was no significant difference in average VAF between the two groups (0.025 vs. 0.028%, respectively; *P* = 0.876 (Wilcoxon rank-sum test); Fig. 3A). All of the mutations identified in D_0_ samples were cleared in the LDC/G-CSF group (Fig. 3B) and the SDC group (Fig. 3C). The clearance pattern for each patient is shown in Supplementary Fig. S3. In a comparison of fusion gene-based minimal residual disease detection with VAF changes of mutations by sequencing, there was relatively high agreement between the two methods (Supplementary Fig. S3).

We further examined mutation clearance of leukemia-associated recurrent mutated genes (RMGs) (Supplementary Table S6), as reported previously [19–21]. A previous study found that different genes might respond differently to induction chemotherapy in adult AML [19], for example, epigenetic regulators such as DNMT3A or TET2 are likely to persist during clinical remission, whereas somatic mutations that activate signaling pathways are usually cleared. Interestingly, although mutation clearance in the LDC/G-CSF group tended to be delayed, recurrent signaling pathway-related mutations KIT and NRAS were cleared after induction I irrespective of treatment group, except one NRAS mutation harbored in patient P2 (Fig. 3D and E). Notably, one patient (P18) in the LDC/G-CSF group carried a mutation in epigenetic regulator KDM6A, which had been cleared after induction I (Fig. 3D).

Of all mutations detected in D_0_ samples, only one mutation remained in follow-up analyses by targeted deep sequencing (Fig. 3F). This persistent mutation (*CACNA1G*; VAF, 1.1%) was detected during the 11-month off-therapy visit of a child receiving SDC; this patient relapsed 4 months later. BM samples from 19 CR patients during follow-up (0–19 months off therapy) harbored no detectable mutations (VAF < 1%). For 3/20 patients for whom samples were collected after HCT, no mutation was detectable 6–19 months after transplantation.

## DISCUSSION

In our study, the CR, EFS, OS and CIR rates of 46 children with AML initially given a low-intensity regimen for the first induction remission were comparable to those of 94 children admitted to the same hospital during the study period and given the SDC regimen. Moreover, chemotherapy dose during induction was not associated with CR, EFS, OS or CIR. AML risk of relapse and HCT were independently associated with outcome. Given that ∼90% of relapses in pediatric AML occur within 2 years of diagnosis [22], our results suggest that patients receiving LDC/G-CSF or SDC for remission induction appear to have similar outcomes.

Observed clinical outcomes were supported by mutation clearance patterns during and after completion of therapy. Residual AML detected by next-generation sequencing is an independent prognostic factor and predictor of relapse in AML [19,23], and thus provides a measure of the remission quality. In our study, molecular remission in children attaining complete response with a first course of LDC/G-CSF and then a second course of the same regimen was comparable with that of children receiving two courses of SDC. Targeted sequencing revealed no difference in resurgence of mutations during follow-up, irrespective of the treatment regimen during induction remission.

Intensive chemotherapy regimens for pediatric AML are associated with high morbidity and mortality [24]. Intensifying the induction courses can decrease CIR rates but not improve OS [13,25–27]. Patients receiving intensive induction courses develop severe infections and many need prolonged hospitalization [28]. In many regions of the world, toxicity and costs associated with remission induction make treatment prohibitive [29,30]. In contrast, patients treated with this LDC/G-CSF regimen receive only a two-drug regimen with 10- and 2-times lower doses of cytarabine and anthracycline, respectively, than those given standard AML remission induction regimens. Remarkably, children receiving LDC/G-CSF attained hematologic remission without prolonged myelosuppression. In 34.3% of patients receiving LDC/G-CSF, ANC never dropped <0.5 × 10^9^/L during induction I. Also, the frequency of severe infections in children given LDC/G-CSF was significantly lower than for children given SDC. This finding is unexpected, because 28.2% of patients treated with LDC/G-CSF because they had active infection and were not candidates for SDC. Infection was managed simultaneously with AML treatment and no patient died from toxicity. Reduced duration of neutropenia and thrombocytopenia, and fewer infectious complications, suggest that the integrity of the hematopoietic system was preserved during hematologic remission in children receiving LDC/G-CSF. These observations challenge the concept [31] that near-myeloablation is required for complete and durable remission.

Although mechanisms underlying the clinical efficacy of LDC/G-CSF are currently unknown, it is possible that G-CSF makes leukemia cells more vulnerable to chemotherapy through sensitization or priming [32,33]. G-CSF can also reduce the overall viability of AML cells when cocultured with BM stroma but not alone [34]. These observations are consistent with findings that resistance of leukemia cells to cytarabine appears to be, in part, mediated by interactions between leukemia cells and BM stroma [35,36]. Finally, G-CSF mobilizes T-regulatory cells and has other immunomodulatory effects [37,38]. Such changes in immune regulation can be exploited by immunotherapy for AML. In some trials, G-CSF/GM-CSF can accelerate BM recovery, and thereby reduce morbidity and mortality when used immediately after completion of induction chemotherapy [39,40]. However, these trials have not established that G-CSF improves remission rates of AML patients.

Because G-CSF can potentially stimulate leukemia cell proliferation [41], children with WBC counts higher than 70 × 10^9^/L did not receive LDC/G-CSF in our study. We found no evidence of increased expansion of leukemia cells in the peripheral blood of children receiving LDC/G-CSF during first induction remission (data not shown). Importantly, as the initial WBC count is considered to be one of the most important influencers on the outcome of AML, we added initial WBC as covariates and found that chemotherapy dose was not significantly associated with OS or EFS in our cohort. Also, the OS and EFS were not significantly different between the LDC/G-CSF group and a SDC subgroup with WBC count <70 × 10^9^/L. Nevertheless, the efficacy of the LDC/G-CSF regimens in pediatric AML patients with hyperleukocytosis remains to be investigated.

Our initial rationale for offering a low-intensity regimen for children with AML was based on the high risk of toxicity-related death or treatment abandonment due to economic reasons. Hence, our results have major implications for children in limited-resource countries. Furthermore, low-intensity treatment as induction remission therapy might impart a reduced selection pressure on the leukemic genome and clonal structure, while use of G-CSF will concurrently sensitize leukemic cells to achieve efficient mutation clearance. However, our study has some limitations including that it was non-randomized, recruited a relatively small number of patients (*n* = 46) and possible selection bias (WBC counts <70 × 10^9^/L). Randomized multicenter clinical trials are needed to further investigate the clinical efficacy of the LDC/G-CSF regimen as induction remission therapy.

## CONCLUSIONS

Pediatric patients with AML treated with LDC/G-CSF during induction had comparable CR, EFS and OS rates, but much lesser toxicity than those treated with SDC. Targeted deep sequencing showed that the quality of remission was also similar between both cohorts. Our findings suggest that hematologic remission can be attained in pediatric AML patients without severe myelosuppression, and that leukemia cell reduction can occur simultaneously with normal hematopoietic regeneration.

## PATIENTS AND METHODS

### Patients

This non-randomized cohort study included children (aged <15 years) with newly diagnosed AML, as defined by WHO criteria [42]. The study was approved by the ethics committee of the Affiliated Children's Hospital of Soochow University. Informed consent was obtained from parents or guardians.

### Diagnosis and risk assignment

AML diagnosis was based on morphologic, immunophenotypic, karyotyping and molecular genetic studies. Patients with promyelocytic leukemia, AML arising after the diagnosis of myelodysplastic syndrome, treatment-related AML or AML of Down syndrome were not included in this study. AML was classified as low, intermediate or high risk according to National Comprehensive Cancer Network criteria [43].

### Treatment

The LDC/G-CSF regimen comprised cytarabine and mitoxantrone or homoharringtonine concurrently administered with G-CSF. The SDC regimen comprised cytarabine, mitoxantrone or homoharringtonine, and etoposide at standard doses (Table 2 and Supplementary Fig. S4). Patients with WBC counts >70 × 10^9^/L were not eligible for LDC/G-CSF. Post-remission therapy was the same for all patients and administered in the following sequence: (1) cytarabine and mitoxantrone, (2) etoposide and cytarabine, and (3) high-dose cytarabine and L-asparaginase (Table 2 and Supplementary Fig. S4). The management of central nervous system leukemia is described in the Supplementary Appendix. Patients at high risk of relapse underwent HCT. Conditioning regimens and prophylaxis for graft vs. host disease have been reported [44], and are summarized in Supplementary Table S7.

**Table 2.** Children's Hospital of Soochow University pediatric acute myeloid leukemia chemotherapy regimens.

	Drug	Dose	Schedule	Days
**Induction**				
LDC/G-CSF	Cytarabine	10 mg/m^2^	Every 12 h, subcutaneous	1–10
	Homoharringtonine^a^	1 mg/m^2^	Once a day	1–7
	Or			
	Mitoxantrone	5 mg/m^2^	Once a day	1, 3, 5
	G-CSF	5 μg/kg	Once a day, subcutaneous	1–10
SDC	Cytarabine	100 mg/m^2^	Every 12 h	1–10
	Etoposide	100 mg/m^2^	Once a day	1–5
	Homoharringtonine^a^	3 mg/m^2^	Once a day	1–7
	or			
	Daunomycin	50 mg/m^2^	Once a day	2, 4, 6
**Post-remission**				
Consolidation I	Cytarabine	2 g/m^2^	Every 12 h	1–3
	Mitoxantrone	10 mg/m^2^	Once a day	3–5
Consolidation II	Cytarabine	3 g/m^2^	Every 12 h	1–3
	Etoposide	150 mg/m^2^	Once a day	1–3
Consolidation III	Cytarabine	3 g/m^2^	Every 12 h	1, 2, 8, 9
	l-asparaginase	6000 U/m^2^	Once a day, intramuscular	3, 10
Consolidation IV	Cytarabine	2 g/m^2^	Once a day	1–5
	Fludarabine	30 mg/m^2^	Once a day	1–5
	G-CSF	300 μg/m^2^	Once a day, subcutaneous	0–5

G-CSF: granulocyte colony-stimulating factor; LDC/G-CSF: low-dose chemotherapy concurrent with G-CSF; SDC: standard-dose chemotherapy.

^a^Mitoxantrone (*n* = 22 patients) and daunomycin (*n* = 16 patients) substituted for homoharringtonine.

### Response evaluation, cost estimation and toxicity monitoring

Response was evaluated after each course of induction chemotherapy. CR was defined as WBC ≥ 1.0 × 10^9^/L, ANC ≥ 0.5 × 10^9^/L, platelet count ≥ 50.0 × 10^9^/L and BM with <5% leukemia cells by morphologic examination. PR was defined as more than 5% and less than 20% of leukemia cells, and NR was defined as 20% or more leukemia cells in the bone marrow. Responses were measured on day 26 of each induction chemotherapy course. Measurement of minimal residual disease by flow cytometry after induction courses was not performed. The Supplementary Appendix provides additional information on response evaluation and the cost of each induction course. Toxicity was evaluated after each treatment course and graded using WHO classification criteria [45].

### Targeted deep sequencing

Targeted deep sequencing of mutations detected at diagnosis was performed in 9 patients who had received LDC/GCSF for induction I and II, and 11 patients who had received SDC for both induction courses. These 20 patients were selected based on sample availability. Sampling time points for sequencing samples are given in Supplementary Fig. S5. The sequencing data reported in this paper were deposited into the Genome Sequence Archive at the BIG data center, Beijing Institute of Genomics, Chinese Academy of Sciences under accession number CRA000956 (http://bigd.big.ac.cn/gsa/s/kwgoQ6mK).

### Statistical analyses

Exact Pearson's chi-squared test was used to compare categorical variables between two or more groups. Wilcoxon rank-sum test was used to compare continuous variables between two groups. Risk group was used in survival analyses as a stratification factor or a covariate. Kaplan–Meier analysis was used to estimate survival functions. Stratified log-rank test and Cox regression model were applied to compare OS and EFS between the LDC/G-CSF and SDC groups. CIR or refractory AML was estimated by the Kalbfleisch–Prentice method, accounting for competing risk [46]. Gray's test and the Fine–Gray regression model were applied to compare cumulative incidence of relapses between both groups. All tests were two-sided. *P*<0.05 was considered statistically significant. Time to failure is defined in the Supplementary Appendix.

## Supplementary Material

nwz006_Supplemental_File
