# Supplementary material for: Ecological principle meets cancer treatment: treating children with acute myeloid leukemia with low-dose chemotherapy
Source: Natl Sci Rev. 2019 Jan 22;6(3):469–79. doi: 10.1093/nsr/nwz006 (PMC8291445; doi:10.1093/nsr/nwz006)
Supplement: nwz006_Supplemental_File [file nwz006_supplemental_file.docx]

**Supplementary Online Content**

**Ecological Principle Meets Cancer Treatment: Treating Children with Acute Mye-loid Leukemia with Low-Dose Chemotherapy**

Yixin Hu, MD^1,7^, Aili Chen, PhD^2,7^, Xinchang Zheng, BS^2,6,7^, Jun Lu, MD^1,7^, Hailong He, MD^1^, Jin Yang, MD^1,5^, Ya Zhang, BS ^2,6^, Pinpin Sui, BS ^2,6^, Jingyi Yang, MD^2,6^, Fuhong He, PhD^2^, Yi Wang, MD, PhD^1^, Peifang Xiao, MD^1^, Xin Liu, MD, PhD^2,6^,Yinmei Zhou, MS^3^, Deqing Pei, MS^3^, Cheng Cheng, PhD^3^, Raul C. Ribeiro, MD^4,#^ , Shaoyan Hu, MD, PhD^1,#^ and Qian-fei Wang, PhD^2,6,#^

**Supplementary Methods.** Methods and Results.

**Supplementary Figure 1.** Flow Chart of Treatment.

**Supplementary Figure 2.** Survival of Patients with Initial White Blood Cell (WBC) Count<70×10^9^/L.

**Supplementary Figure 3.** Mutational Clearance Detected by Exome and Targeted Sequencing and PCR-Based MRD Detection for Patients.

**Supplementary Figure 4.** Flowchart of AML Treatment Regimens Used for Study Participants.

**Supplementary Figure 5.** Sampling Time Points and Sequencing Methods.

**Supplementary Table 1.** Cox Regression Analysis of the Entire Cohort with AML Risk Group and Treatment Regimen as Covariates.

**Supplementary Table 2.** Cox Regression Analysis of the Entire Cohort with Treatment, Gender, Age, Initial WBC, AML Risk, and HCT as Covariates.

**Supplementary Table 3.** Cox Regression Analysis with Treatment, Gender, Age, Initial WBC, AML Risk, and HCT as Covariates in Patients with Initial WBC Count <70×10^9^/L.

**Supplementary Table 4.** Toxicity and Cost by Treatment Group.

**Supplementary Table 5.** Clinical and Genomic Characteristics at Diagnosis for Patients Whose Samples were Analyzed by Ampliseq.

**Supplementary Table 6.** Leukemia-Associated Recurrent Mutated Genes (n=268) Used in This Study.

**Supplementary Table 7.** Children's Hospital of Soochow University Pediatric Acute Myeloid Leukemia Hematopoietic Cell Transplant Regimens.

**Supplementary Table 8.** Cox Regression Analysis with Treatment, Gender, Age, Initial WBC Count, Final Risk, and HCT as Covariates.

**Supplementary Table 9.** Cox Regression Analysis with Treatment, Gender, Age, Initial WBC, Final Risk, and HCT as Covariates in Patients with initial WBC<70×10^9^/L.

**Supplementary References.**

# Supplementary Methods. Methods and Results.

## 1. Patients, Methods and Results

### 1.1 Response Evaluation

Patients with complete response (CR) received the same regimen used in the first remission no later than day 36. Patients who attained PR also received induction II immediately with the same regimen they received in induction I. Patients with refractory leukemia who had received the LDC/G-CSF regimen for induction I received SDC for induction II. Post-remission therapy varied according to risk classification. Patients at low risk of relapse received 3 courses of consolidation therapy. Patients at high risk of relapse receive consolidation I and then hematopoietic cell transplant. If no suitable donor was available for these patients, they received 4 courses of postremission chemotherapy.

Patient samples with *RUNX1/RUNXT1* or *CBFβ/MYH11* translocations were analyzed by real-time RT-PCR and those with *MLL* rearrangements were analyzed as reported previously [^1^](#_ENREF_1). Flow cytometric evaluation of residual disease was not included in the response evaluation.

### 1.2 Treatment of Central Nervous System Leukemia

Patients with central nervous system (CNS) involvement, defined as the presence of at least five white blood cells per µL in cerebrum spinal fluid (CSF) with leukemic blasts or in patients with abnormal findings on brain imaging, received weekly doses of age-adjusted intrathecal methotrexate, dexamethasone and cytarabine (ITMDA) until the CSF was clear of leukemia (minimum four doses) and then three monthly doses. Patients without CNS involvement received four monthly doses of ITMDA.

### 1.3 Statistical Analysis

For overall survival, failure included death from any cause. For event-free survival (EFS), failure included death, relapse, induction failure, and abandonment. Starting time was the beginning date of induction I; EFS times for patients who had induction failure were set to 0. For cumulative incidence of relapse (CIR), failures included relapse after achieving complete remission (CR) or induction failure. Time to failure for patients with induction failure was set to 0. Death while in CR and abandonment were considered as competing events. Statistical analyses were performed using SAS version V. 9.4, Cytel Studio V. 11, and SPSS V. 16.

### 1.4 Results

After completion of two induction courses, AML was reclassified according to response to therapy. Patients with morphologic evidence of AML, irrespective of the initial AML risk classification, were considered at high risk of relapse (final AML risk classification) and received intensified chemotherapy or HCT. Treatment outcome significantly differed by AML risk group after inductions I and II. In multivariate regression analyses repeated with final AML risk classification as an explanatory variable, chemotherapy dose did not affect relapse risk (Supplementary Tables 8 and 9).

Treatment cost was calculated for each patient based on actual charges for laboratory tests, chemotherapy drugs, anti-infective drugs, and blood transfusions. Median cost for induction I per patient was significantly lower for the LDC/G-CSF group than for the SDC group ($7,463 [range $1,916–20,189] vs. $10,315 [range $3,762–28,926]; *P*<0.001; Supplementary Table 4). Similarly, median cost of induction II per patient was significantly lower for the LDC/G-CSF group than for the SDC group ($2,363 [range $1,332–8,258] vs. $ 5,719 [range $1,748–21,118]; *P*<0.001; Supplementary Table 4).

## 2. Targeted Sequencing and Analysis

### 2.1 Sample Selection

Ion Ampliseq^TM^ sequencing was performed to determine mutational clearance during induction chemotherapy, postremission chemotherapy and follow up. A total of 149 mutations identified by exome sequencing, including 137 found in diagnosis samples and 12 identified in relapsed samples, were analyzed. T cells were isolated by flow cytometry, using a combination of CD45 and CD3 double-positive cells. Genomic DNA was extracted later to serve as normal control.

Samples were available at different time points during the whole treatment and follow up. In total, 86 samples were sequenced, including 7 samples obtained from the bone marrow smear (Supplementary Figure 5).

### 2.2 Library Construction

A bed file with the position of those mutations was submitted to Ion Ampliseq^TM^ Designer version v5.6.3 (<https://www.ampliseq.com/browse.action>) to generate primers, and all amplicons were designed under the DNA single pool workflow.

Libraries were generated following the manufacturer’s Ion AmpliSeq Library Kit 2.0 protocol (Thermo Fisher, Cat. no. 4476610). After AMPureXP bead purification (Beckman Coulter), concentration of the library was determined by qPCR, using the Ion Library TaqMan Quantitation Kit (ThermoFisher, #4468802). Emulsion PCR and enrichment were performed using the Ion PI HI-Q Template OT2 200 Kit (Thermo Fisher, #A26434). Template-positive ISPs were enriched and sequenced using the Ion PI HI-Q Sequencing 200 Kit (Thermo Fisher, #A26433) on Ion Torrent according to the manufacturer’s instructions.

### 2.3 Sequencing and Analysis

Processing of raw data, removal of adapter sequences, base calling, and quality value calculations were performed using Torrent Suite™ Software 4.4. Sequences aligned to genome (Hg19) by using the Torrent Mapping Alignment Program. Initial variant calling was generated using the Ion Torrent platform-specific pipeline software Torrent Variant Caller 4.4 (parameters: --hotspot_min_allele_freq: 0.0015 --outlier_probability: 0.0007 --downsample_to_coverage: 20000 --max_detail_level: 10000).

For targeted deep-sequencing, approximately 20 ng input DNA was used, translating to roughly 3000 diploid cells. Theoretically, the sensitivity limit to detect VAF is 0.03% (1 in 3000 cells). In practice, the sensitivity and performance of an amplicon-sequencing base assay are dependent on primers, type of mutations, and read of distribution in multiplex reactions (copied sequence) Hence, samples were considered to have a mutation if the percentage of mutated reads exceeded 1% (10 times the expected base miscall rate of 0.1%).

# Supplementary Figures

### Supplementary Figure 1. Flow Chart of Treatment.

* Seventeen patients underwent HCT directly after induction II and 41 patients after at least one course of consolidation therapy.

###
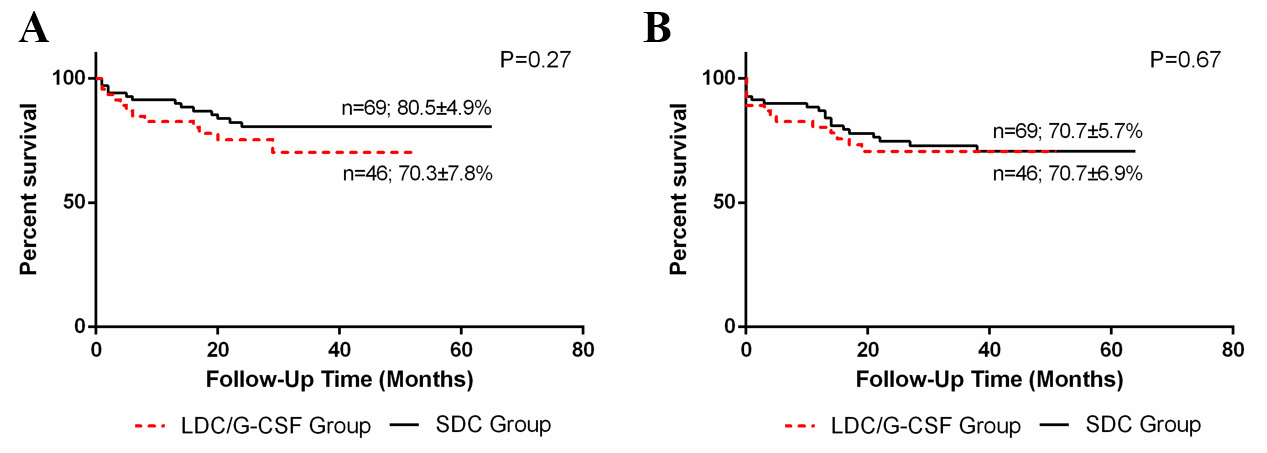
Supplementary Figure 2. Survival of Patients with Initial White Blood Cell (WBC) Count<70×10^9^/L.

**A** OS according to regimen for 115 patients with initial white blood cell (WBC) count<70×10^9^/L; **B** EFS according to regimen for patients with initial WBC<70×10^9^/L.

### Supplementary Figure 3. Mutational Clearance Detected by Exome and Targeted Sequencing and PCR-Based MRD Detection for Patients.

Line charts with different colors showing the VAF change in mutations detected at diagnosis for each CR patient. Each line represents one mutation. Black lines represent the VAF analyzed by exome sequencing, and red lines represent VAF changes in the same group of mutations detected by AmpliSeq deep sequencing. Circle graphs reflect the molecular response detected by PCR-based MRD detection.


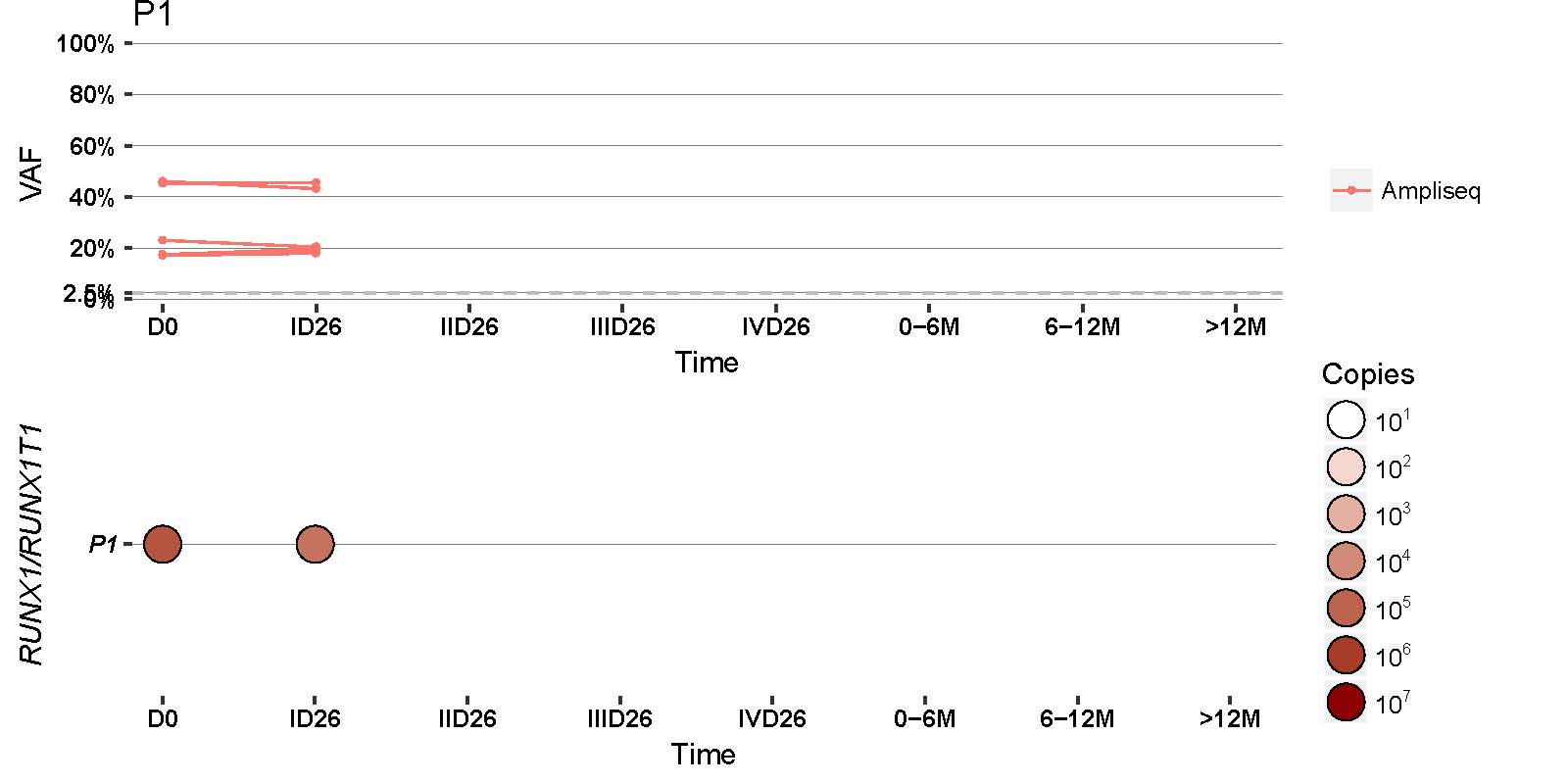


**A**

**B**


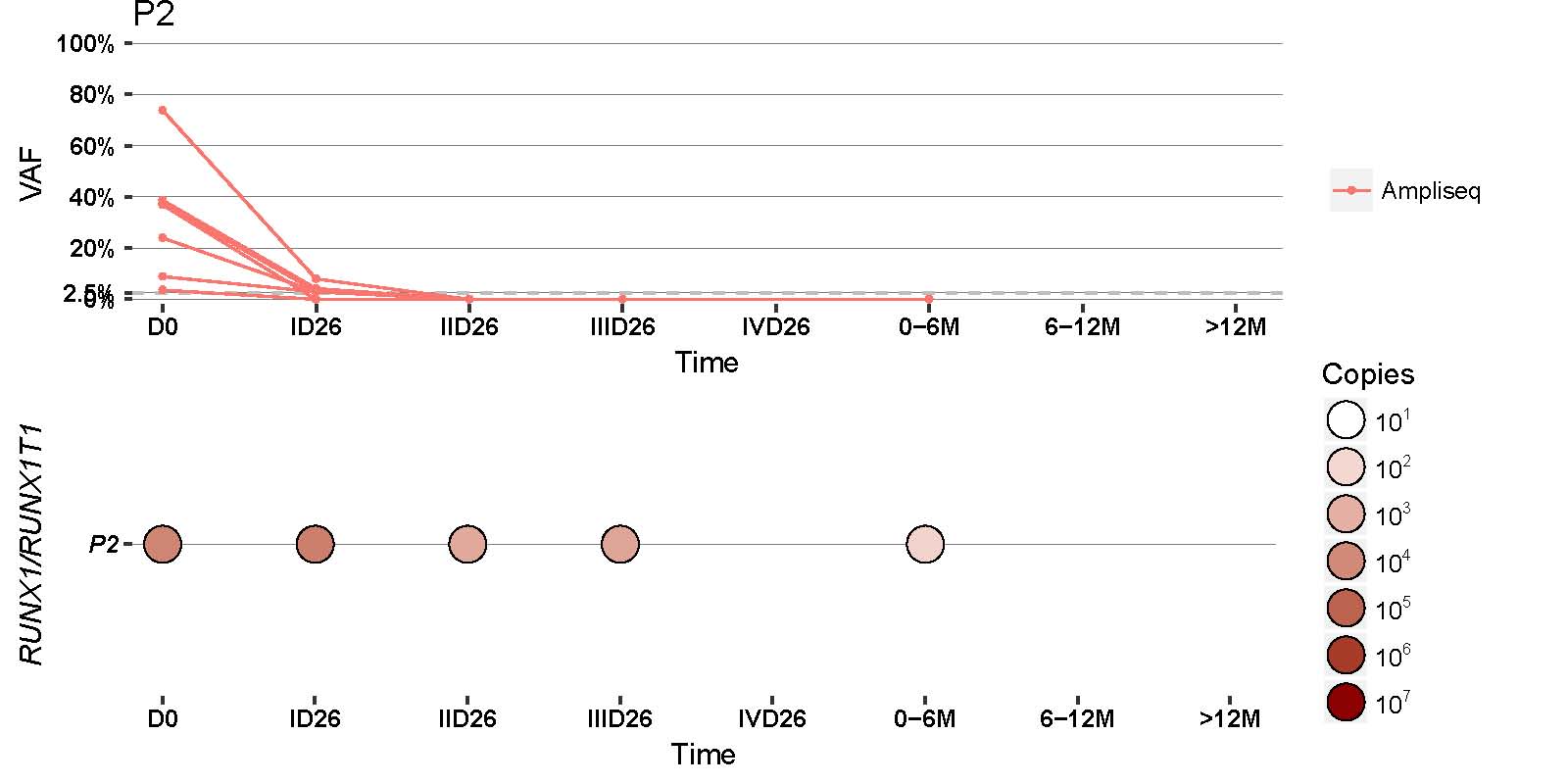


**Supplementary Figure 3.** **Mutational Clearance Detected by Exome and Targeted Sequencing and PCR-Based MRD Detection for Patients (Continued).**

**C**

**D**


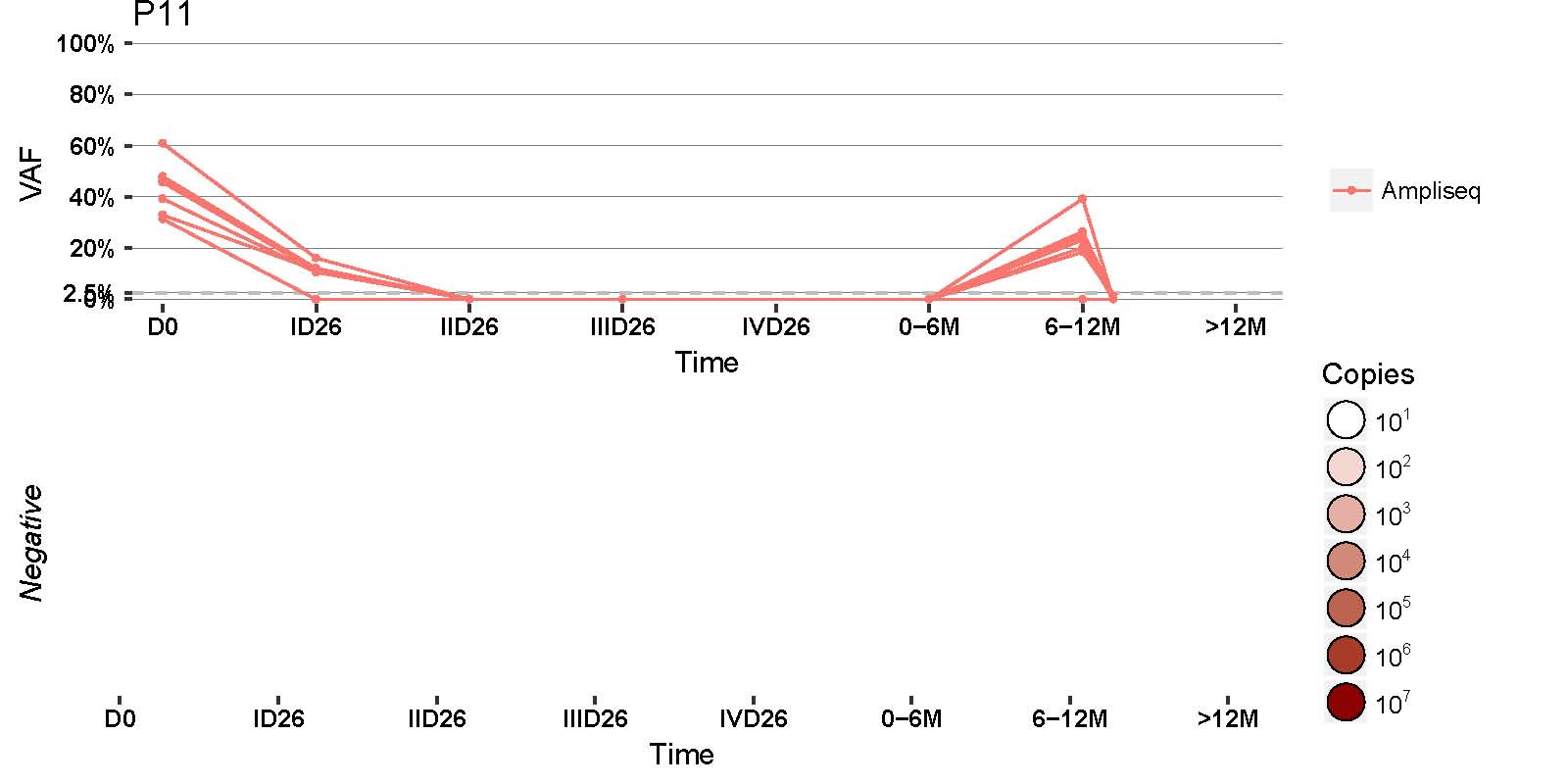


**Supplementary Figure 3. Mutational Clearance Detected by Exome and Targeted Sequencing and PCR-Based MRD Detection for Patients (Continued).**

**E**


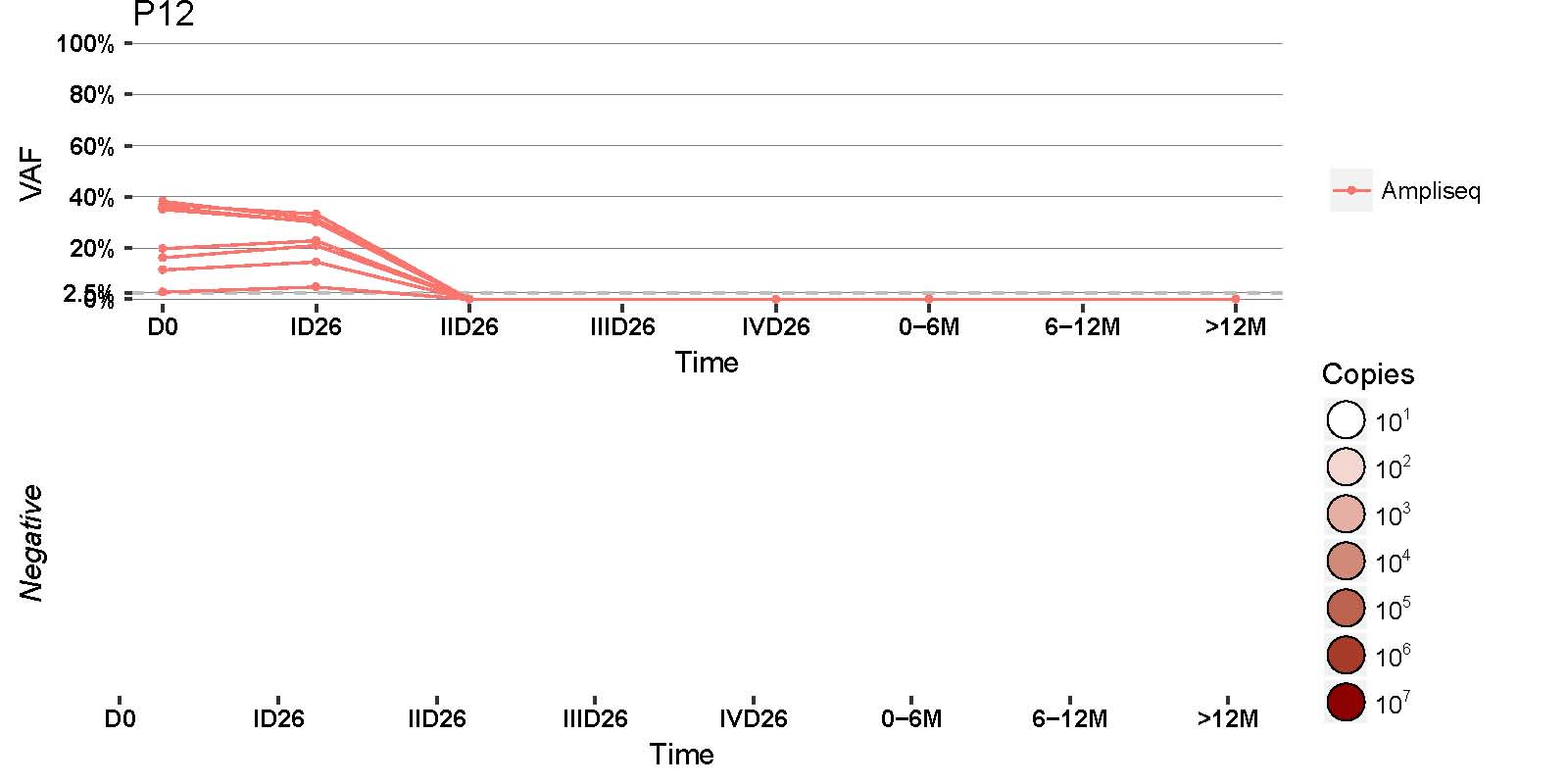

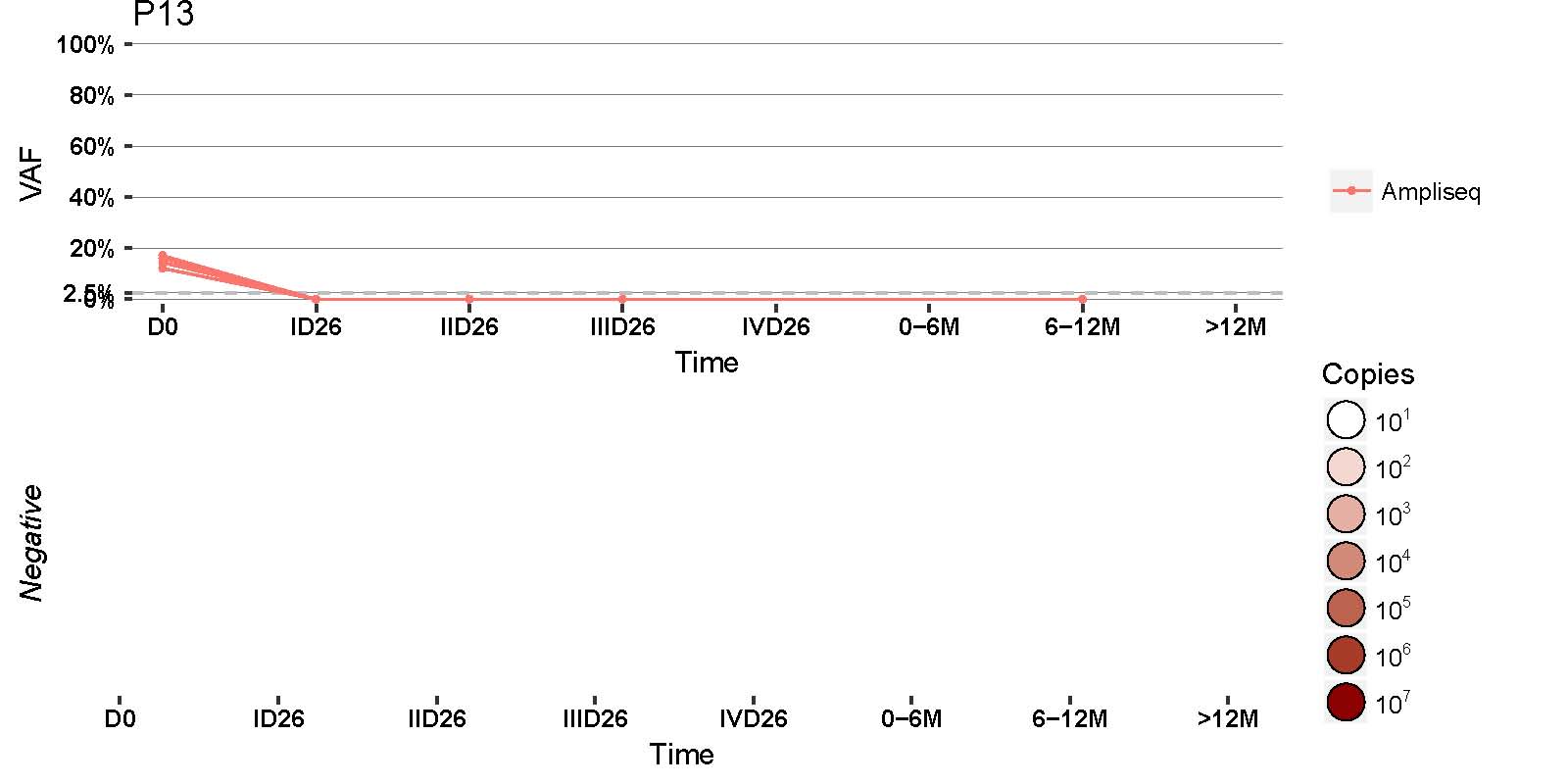


**F**

**Supplementary Figure 3. Mutational Clearance Detected by Exome and Targeted Sequencing and PCR-Based MRD Detection for Patients (Continued).**

**H**

**G**


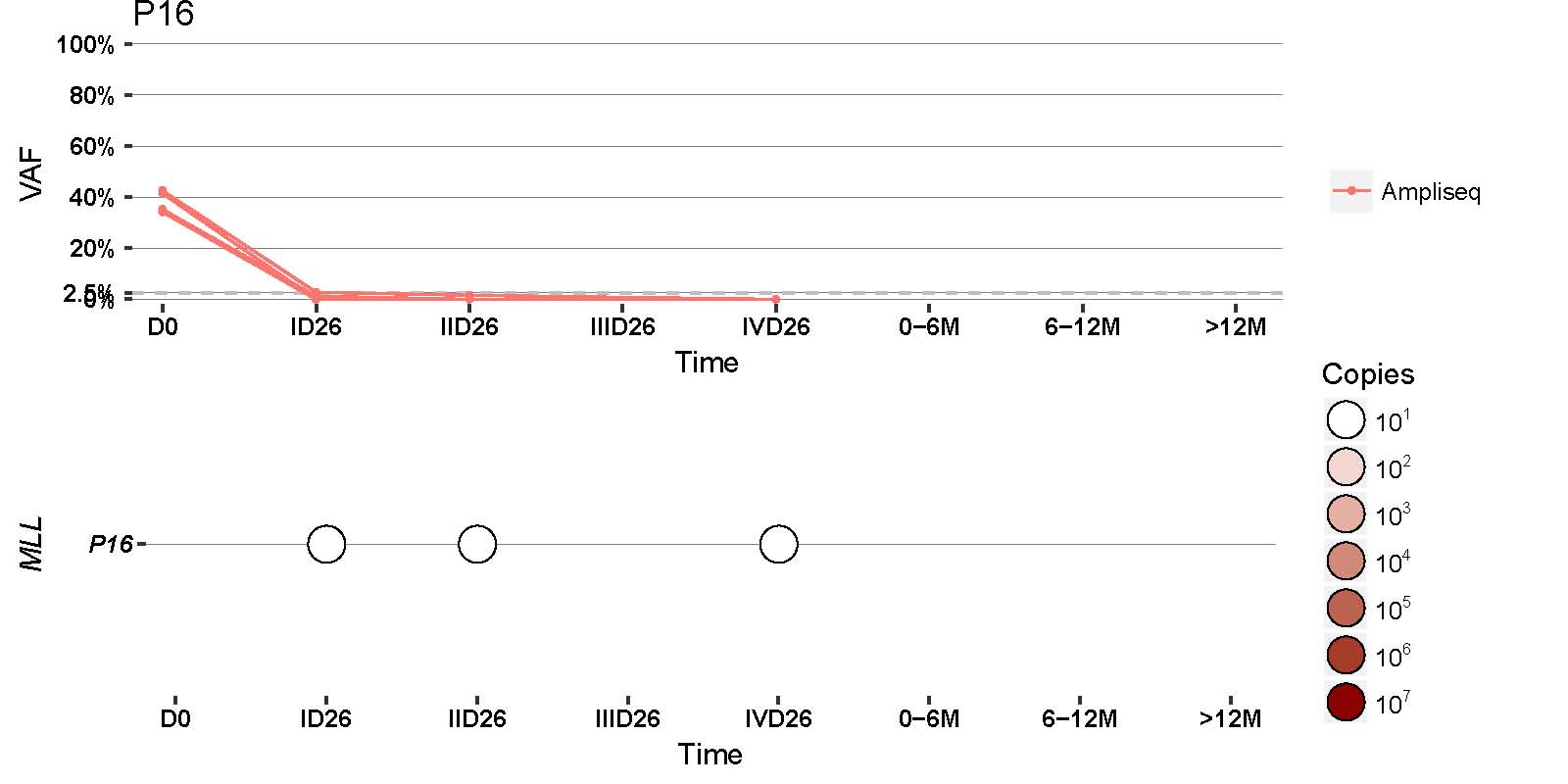

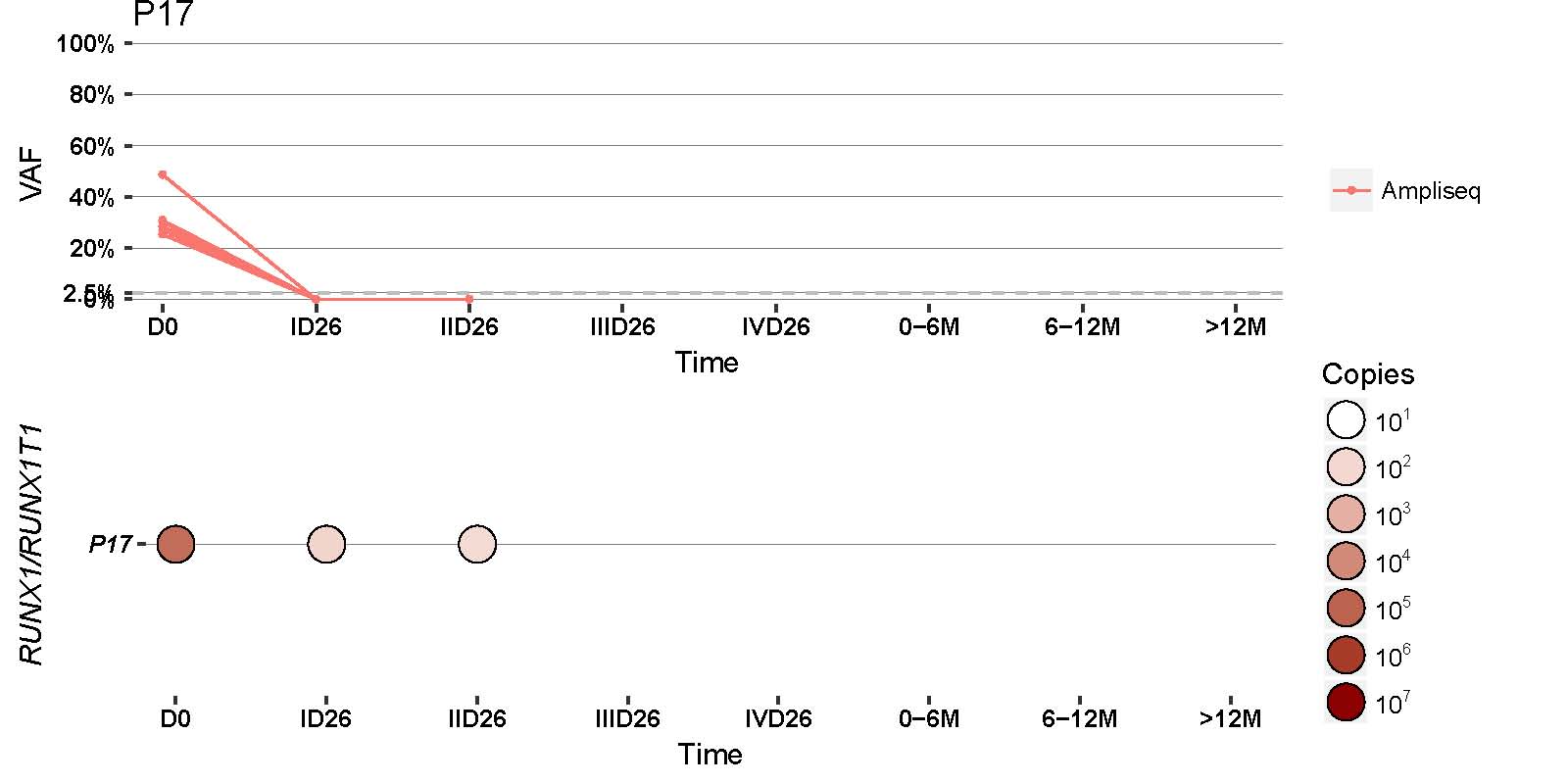

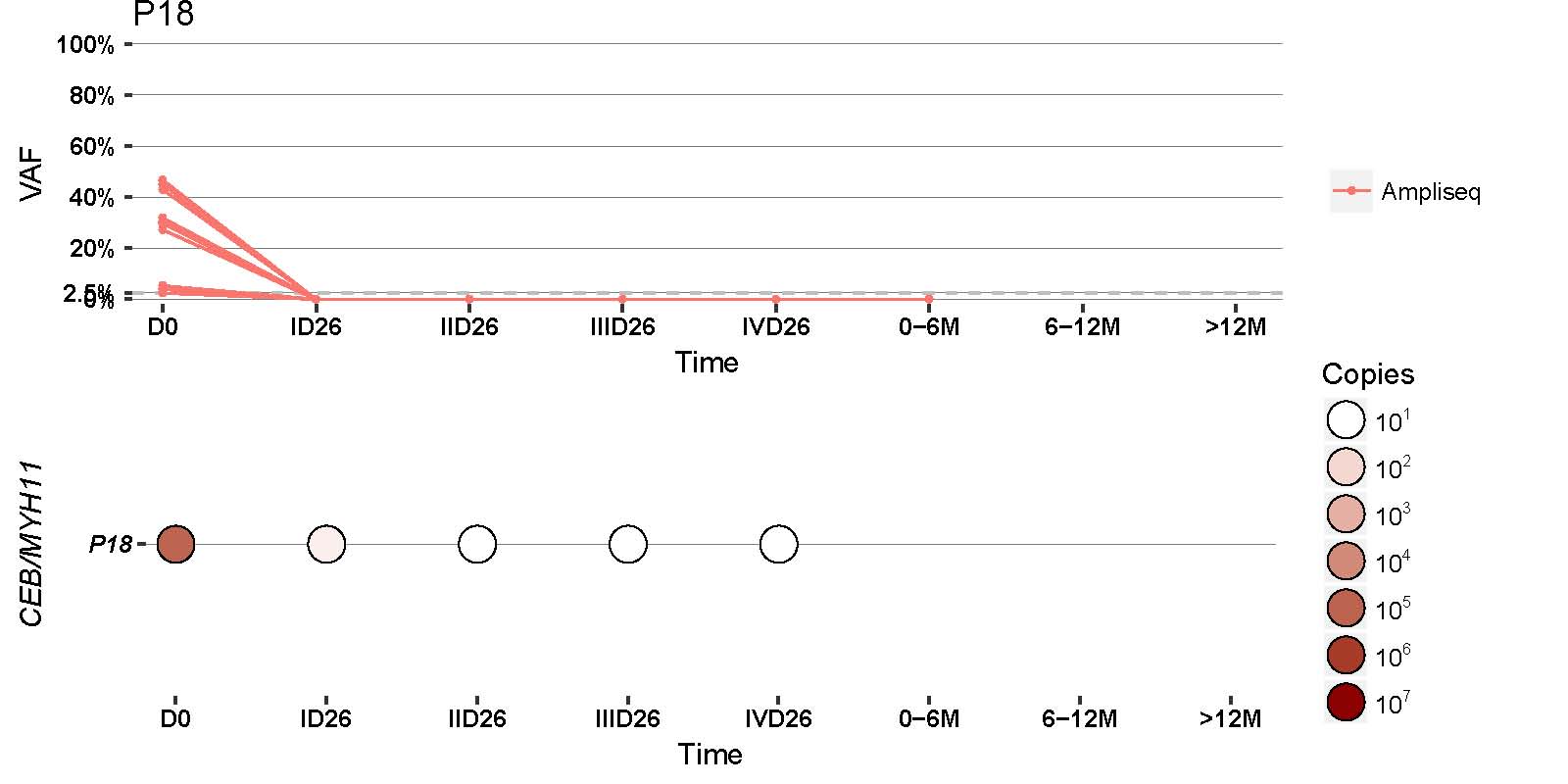


**I**

**Supplementary Figure 3. Mutational Clearance Detected by Exome and Targeted Sequencing and PCR-Based MRD Detection for Patients (Continued).**


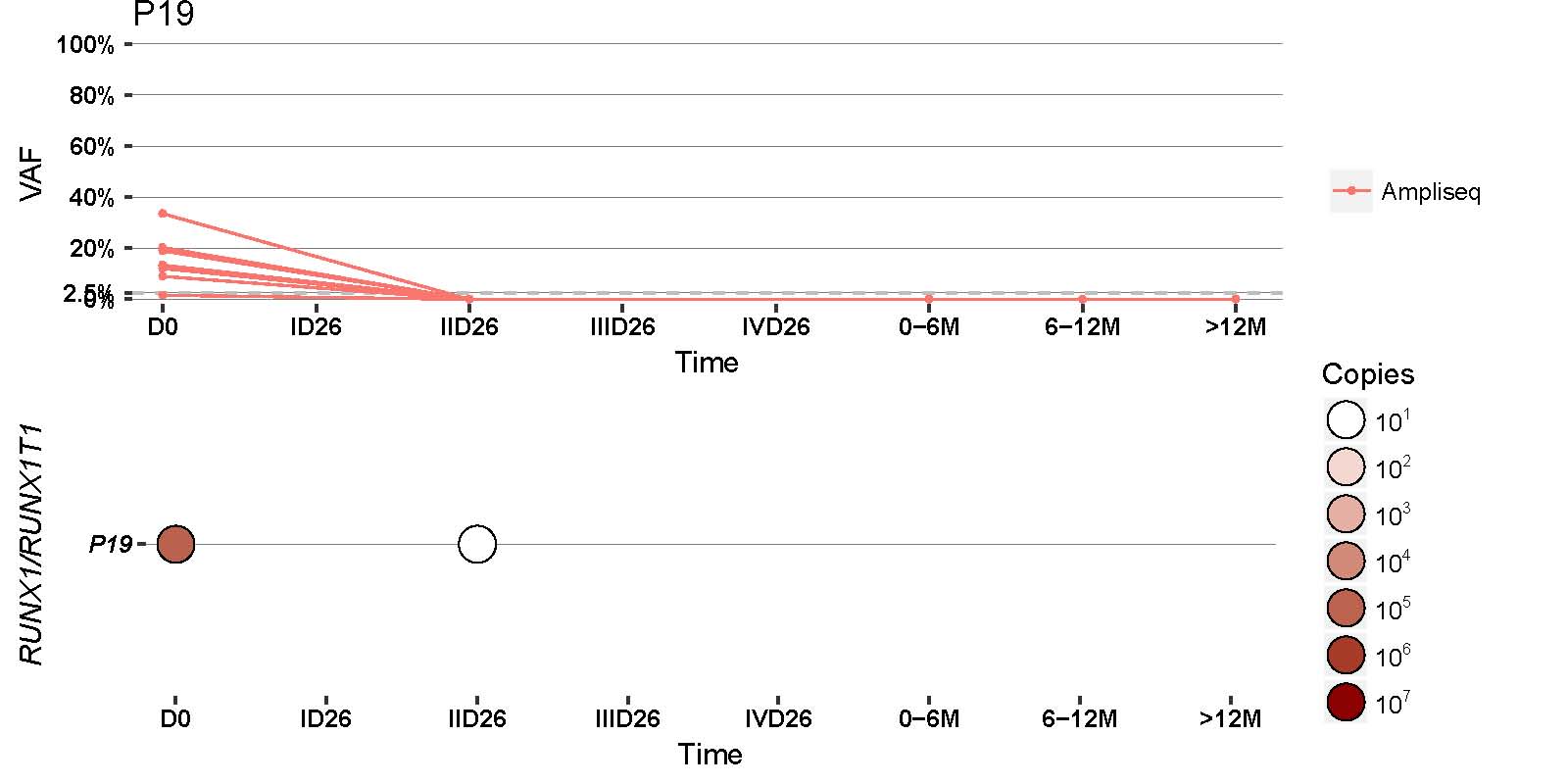

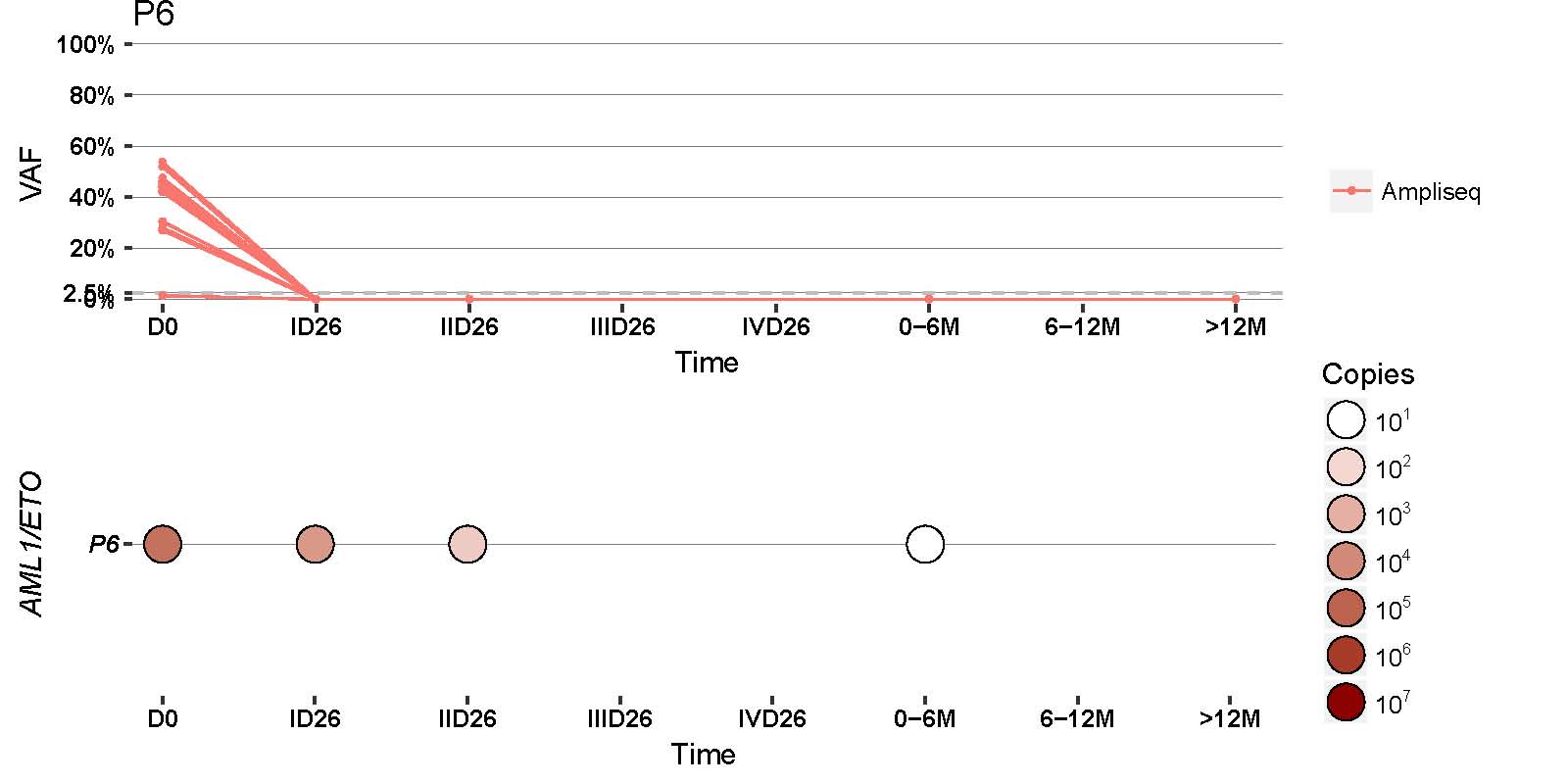


**K**

**J**


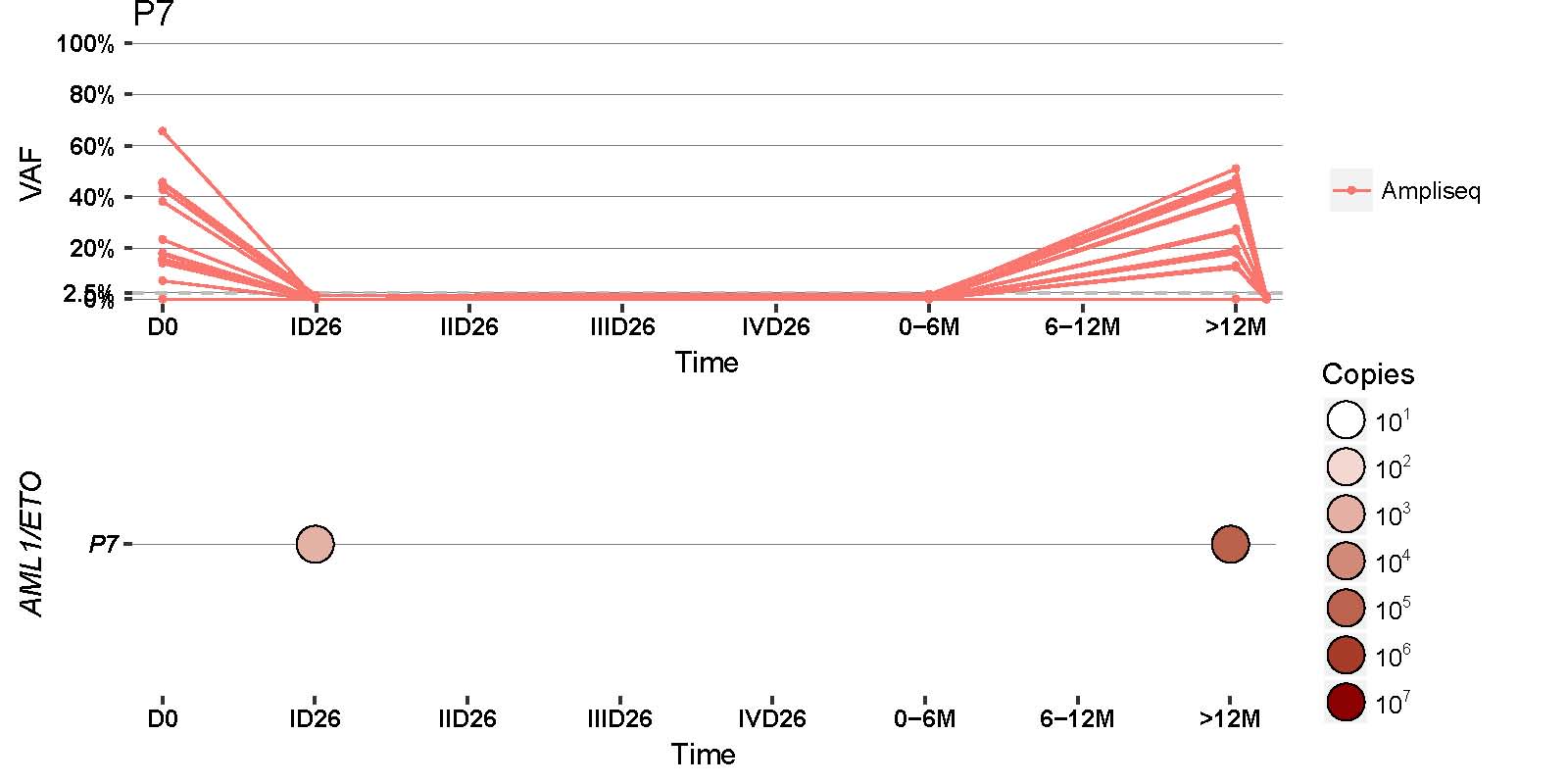


**L**


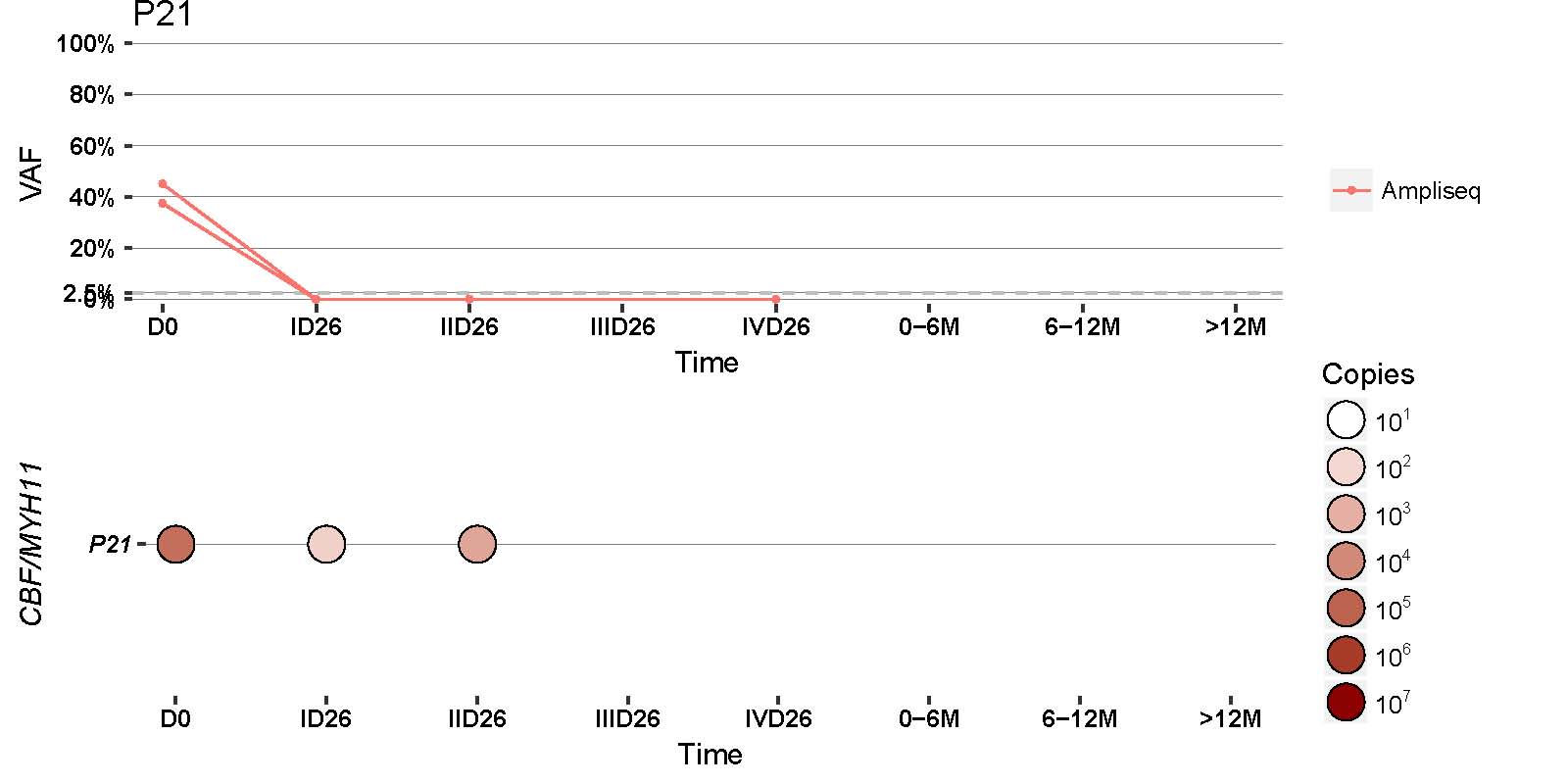

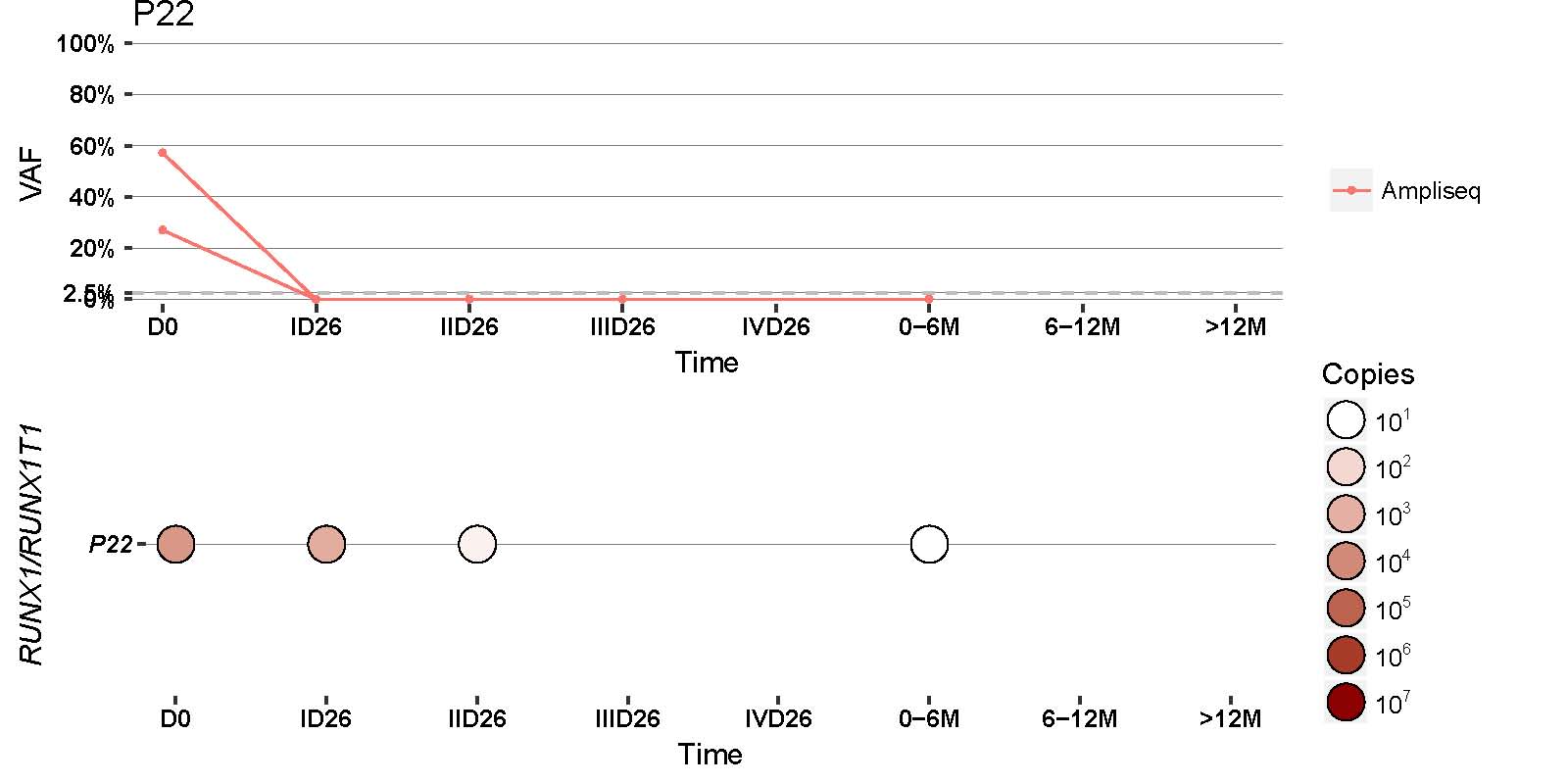


**N**

**M**


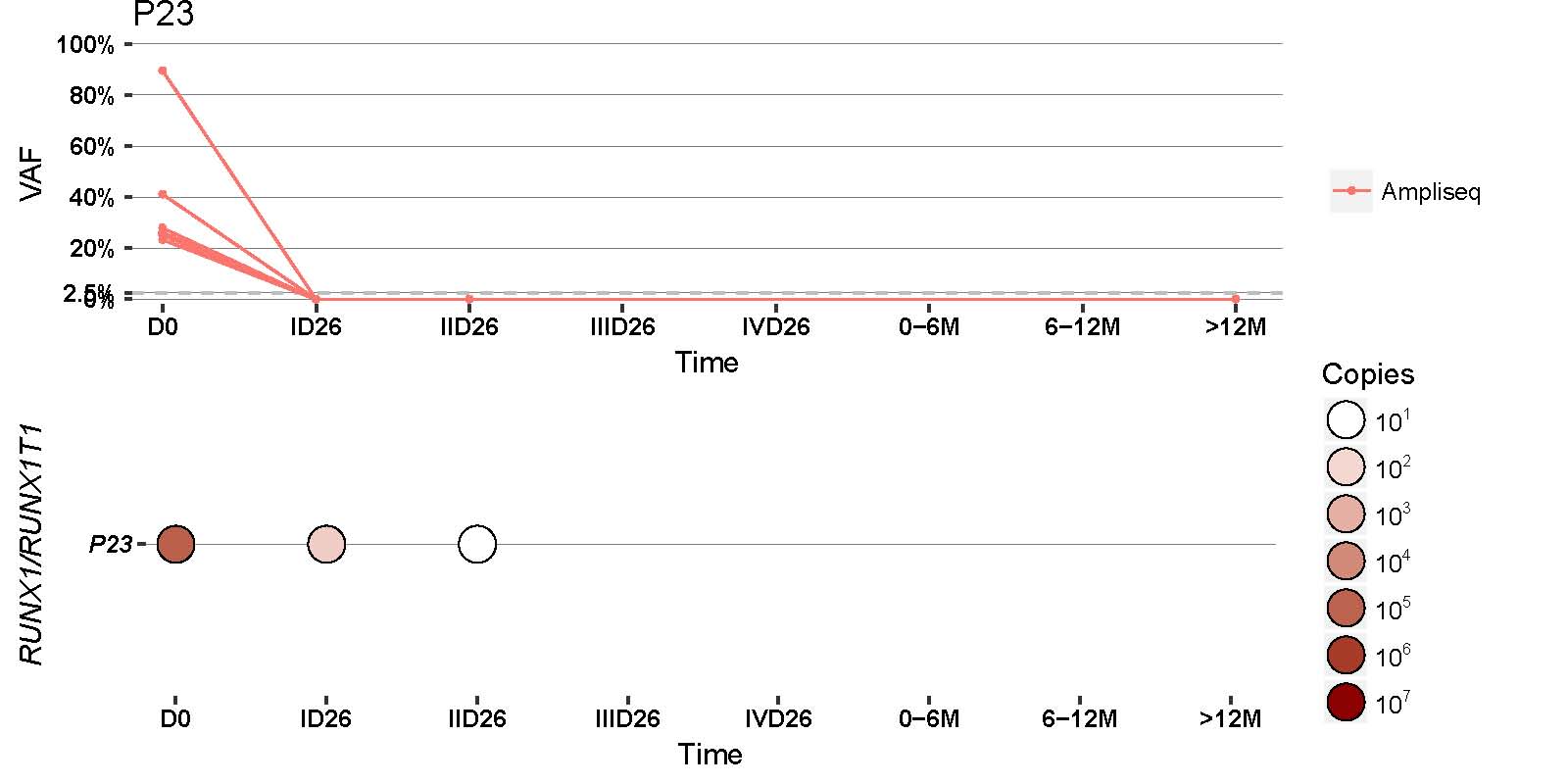


**Supplementary Figure 3. Mutational Clearance Detected by Exome and Targeted Sequencing and PCR-Based MRD Detection for Patients (Continued).**

**O**

**Supplementary Figure 3. Mutational Clearance Detected by Exome and Targeted Sequencing and PCR-Based MRD Detection for Patients (Continued).**


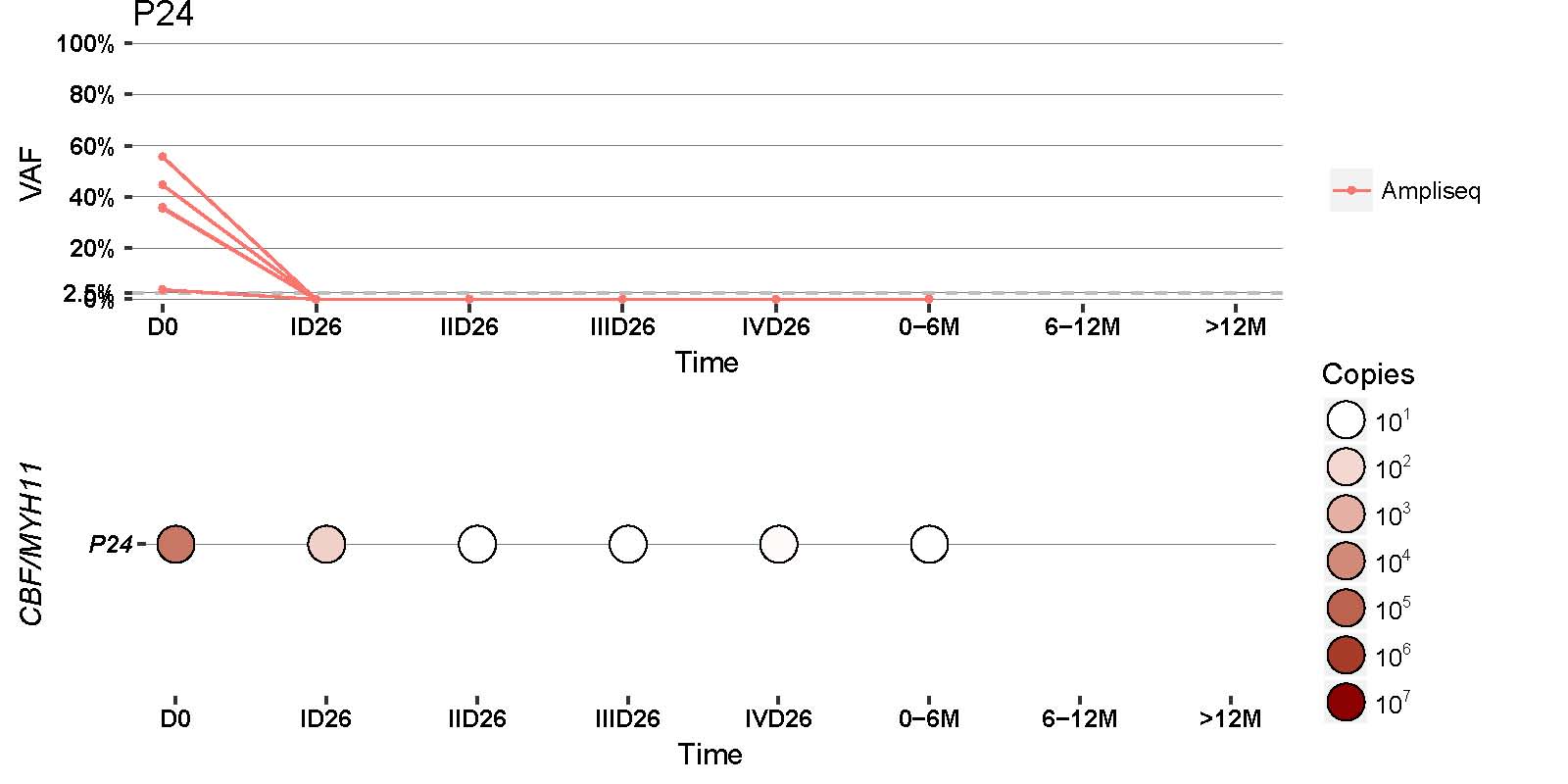

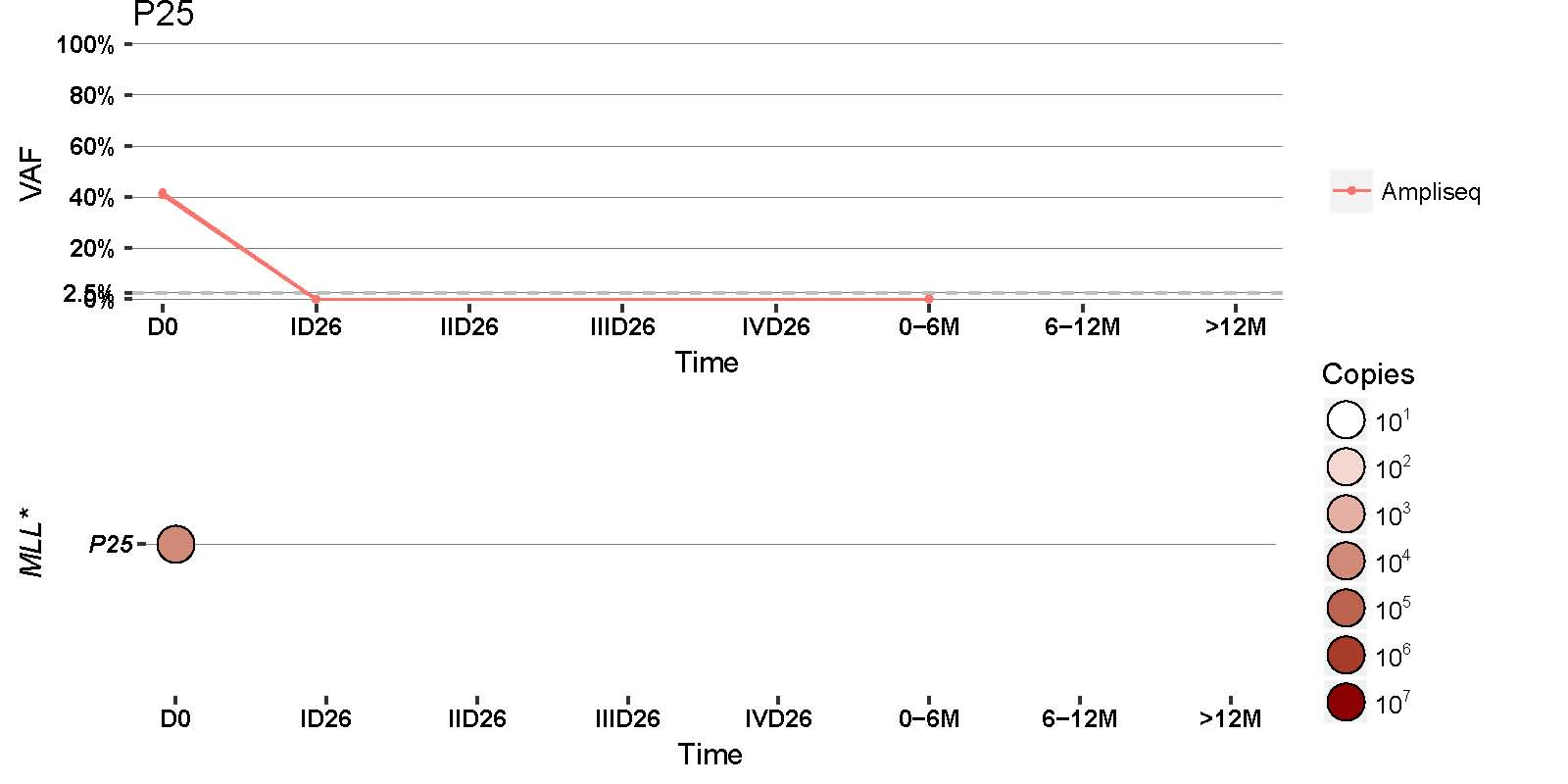


**Q**

**P**


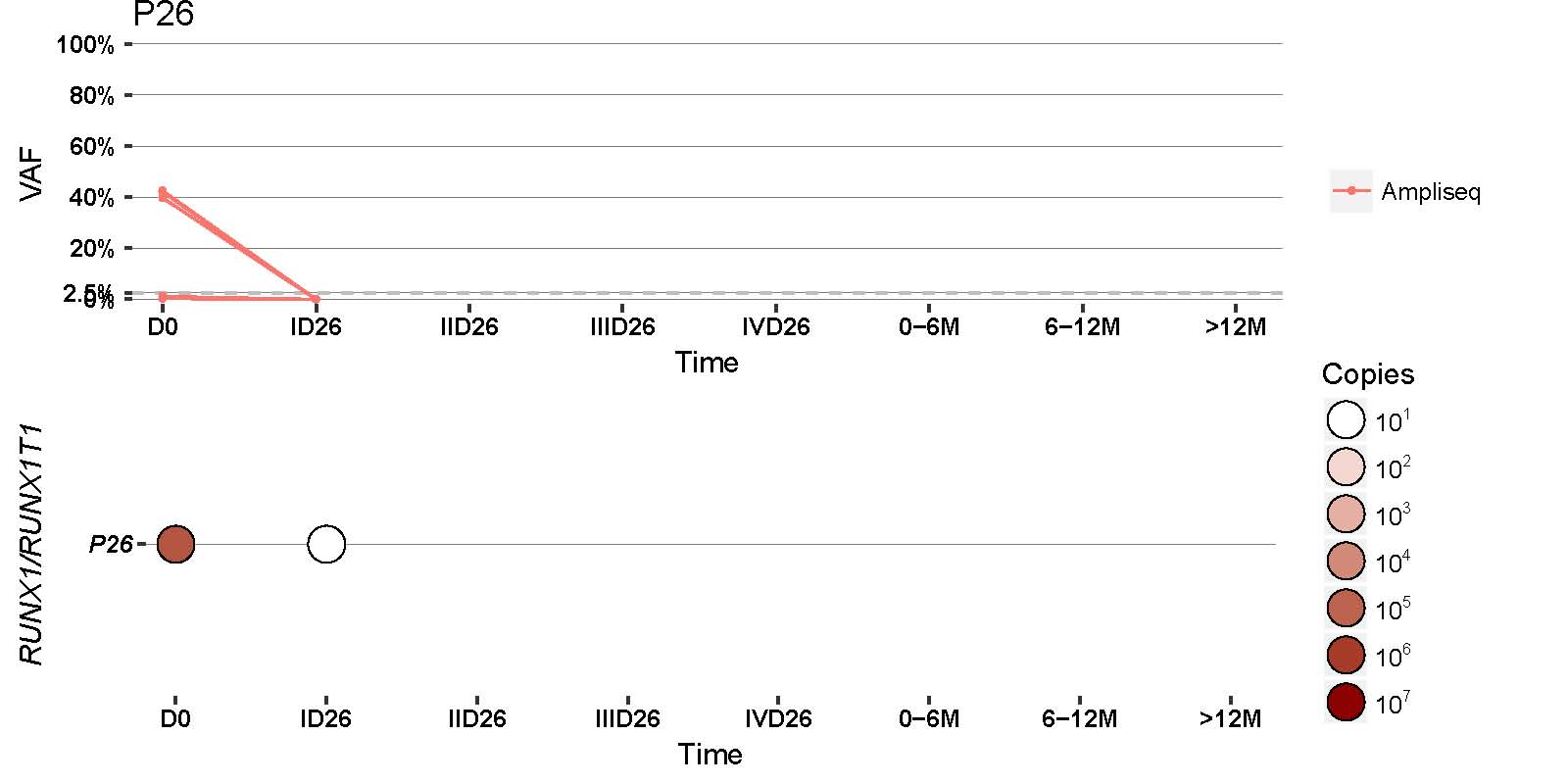


**R**


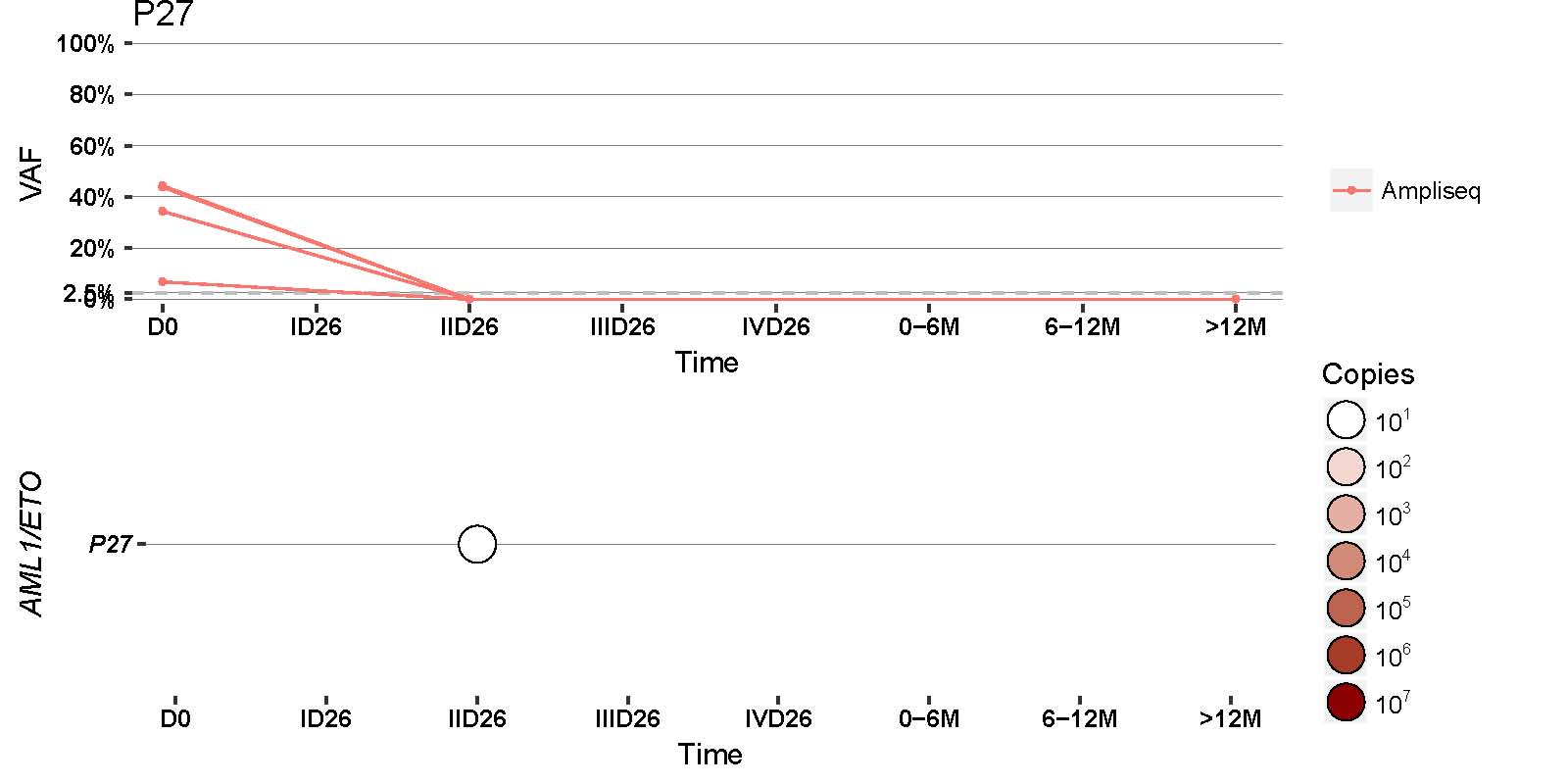

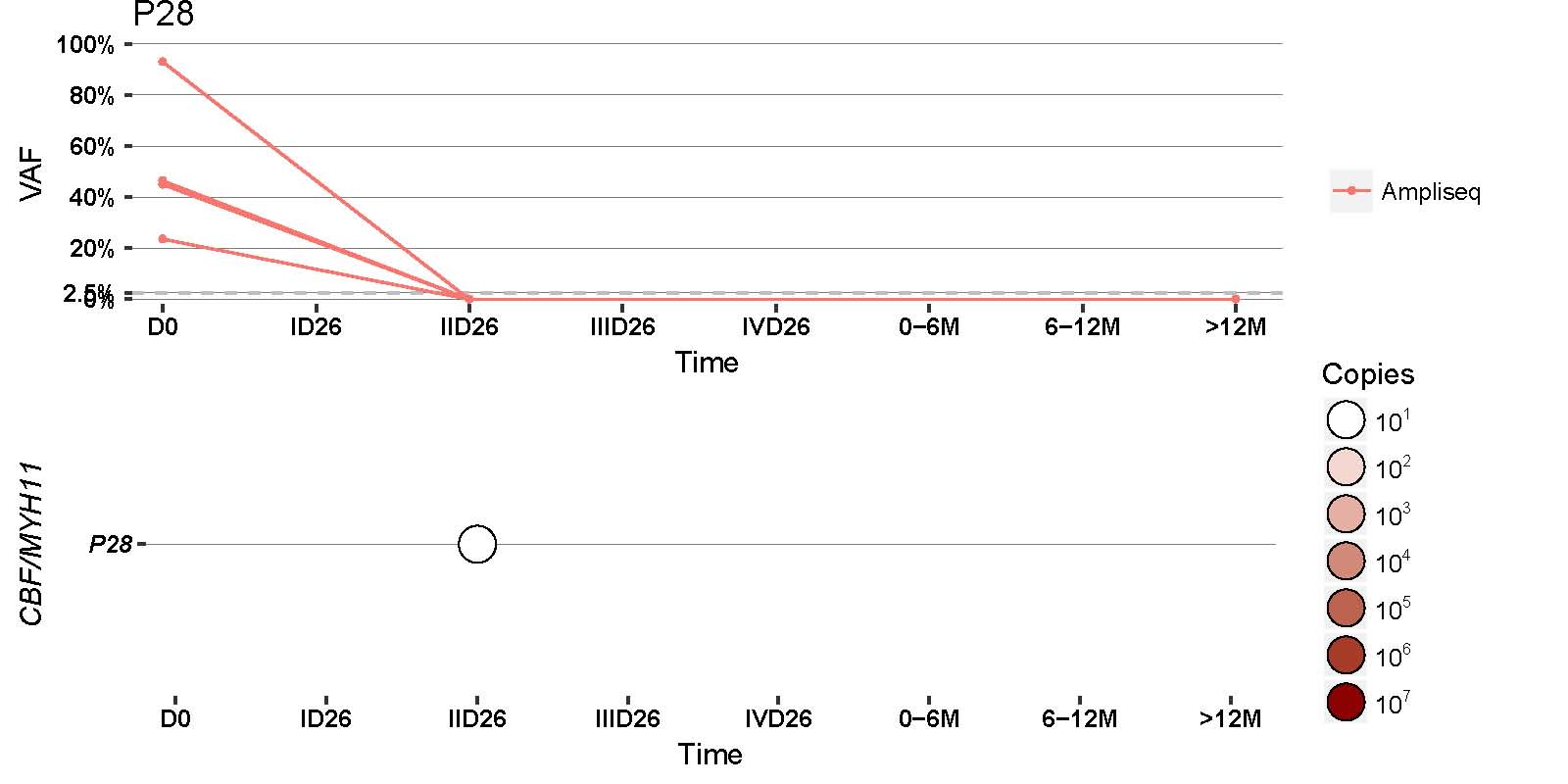


**T**

**S**


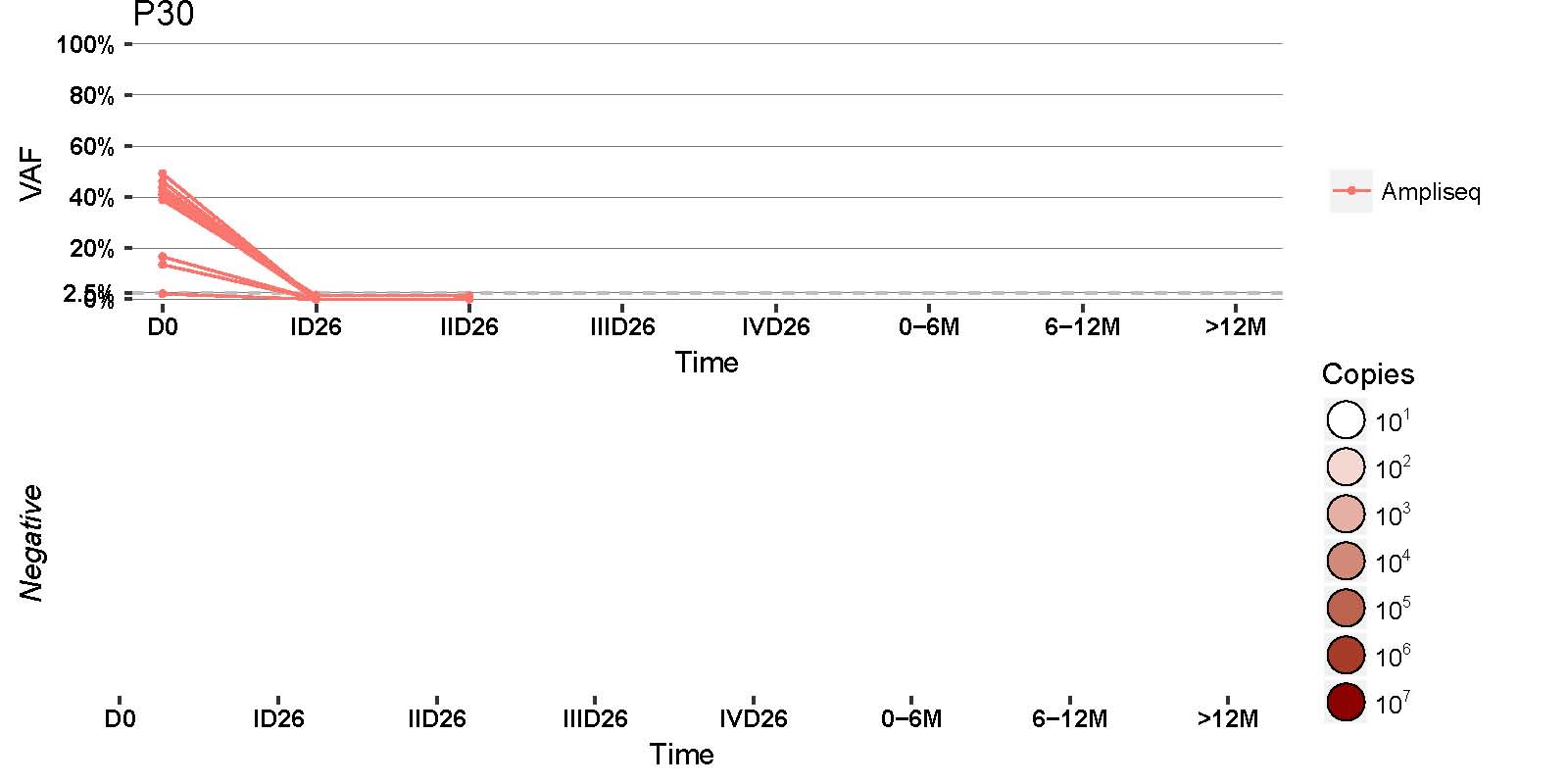


###
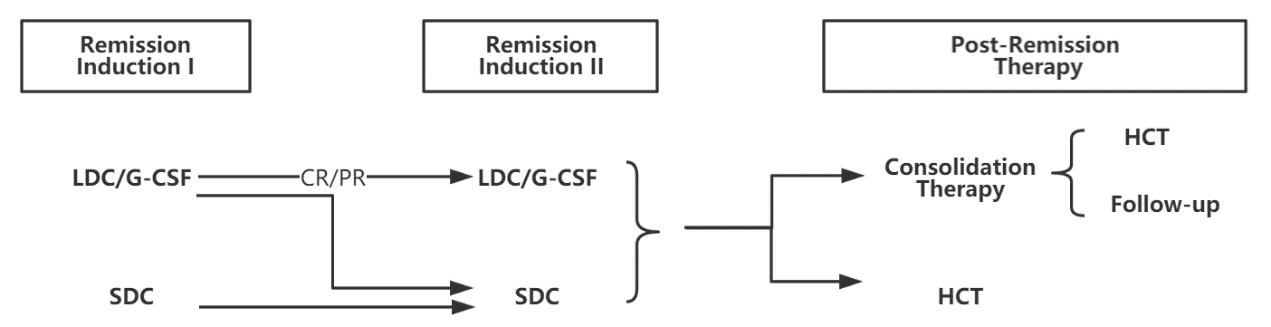
Supplementary Figure 4. Flowchart of AML Treatment Regimens Used for Study Participants.

* Patients were switched to the standard arm if they had ≥25% blasts in the day 26 bone marrow or if the parent or physician requested it.

###
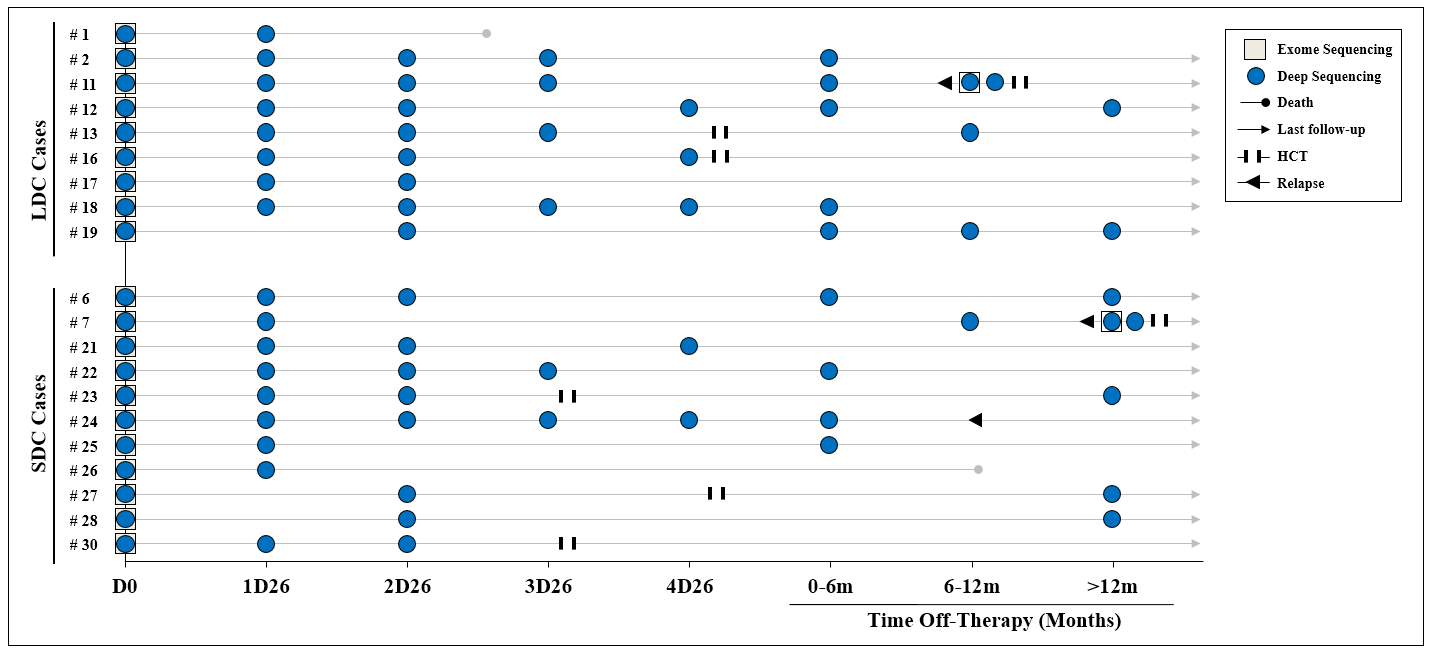
Supplementary Figure 5. Sampling Time Points and Sequencing Methods.

Samples from 20 patients were analyzed. Exome sequencing was performed in 42 samples from 20 patients, including 20 sorted CD3^+^ controls (not shown in the figure) to get the targeted gene list. AmpliSeq targeted deep-sequencing was then performed in 86 samples from 20 patients during the whole therapeutic course, including three samples obtained after HCT, and two pairs of samples obtained at relapse, and after the first chemotherapy course of salvage therapy.

**Supplementary Tables**

### Supplementary Table 1. Cox Regression Analysis of the Entire Cohort with AML Risk Group and Treatment Regimen as Covariates.

| **Clinical Feature** | **EFS** | | | **OS** | | | **Any Relapse** | | |
| --- | --- | --- | --- | --- | --- | --- | --- | --- | --- |
|  | **HR** | **95% CI** | **P value** | **HR** | **95% CI** | **P value** | **HR** | **95% CI** | **P value** |
| AML risk: high vs. low | 4.577 | 1.79-11.7 | 0.0015 | 4.041 | 1.42-11.5 | 0.0089 | 4.368 | 1.55-12.3 | 0.0052 |
| AML risk: intermediate vs. low | 2.748 | 1.09-6.93 | 0.0322 | 2.672 | 0.97-7.36 | 0.0574 | 2.413 | 0.86-6.76 | 0.0937 |
| Treatment: SDC vs. LDC/G-CSF | 1.052 | 0.53-2.09 | 0.8852 | 0.955 | 0.45-2.04 | 0.9054 | 0.918 | 0.43-1.96 | 0.8255 |

Abbreviations: CI, confidence interval; EFS, event-free survival; HR, hazard ratio; LDC/G-CSF, low-dose chemotherapy concurrent with G-CSF; OS, overall survival; SDC, standard-dose chemotherapy.

### Supplementary Table 2. Cox Regression Analysis of the Entire Cohort with Treatment, Gender, Age, Initial WBC, AML Risk, and HCT as Covariates.

| **Clinical Feature** | **EFS** | | | **OS** | | | **Any Relapse** | | |
| --- | --- | --- | --- | --- | --- | --- | --- | --- | --- |
|  | **HR** | **95% CI** | **P value** | **HR** | **95% CI** | **P value** | **HR** | **95% CI** | **P value** |
| Age 1–10 years vs. infant | 1.330 | 0.31-5.79 | 0.7038 | 1.149 | 0.25-5.18 | 0.8561 | 1.947 | 0.26-14.9 | 0.5205 |
| Age >10 years vs. infant | 2.844 | 0.58-14.0 | 0.1977 | 2.835 | 0.55-14.6 | 0.2124 | 3.781 | 0.44-32.5 | 0.2258 |
| WBC 50≤WBC<100 vs. <50 | 0.529 | 0.12-2.28 | 0.3927 | 0.422 | 0.06-3.18 | 0.4022 | 0.685 | 0.16-3.00 | 0.6151 |
| WBC count ≥100 vs. <50 | 2.144 | 0.92-4.97 | 0.0757 | 3.262 | 1.28-8.33 | 0.0134 | 1.839 | 0.69-4.90 | 0.2231 |
| AML risk: high vs. low | 5.944 | 2.17-16.3 | 0.0005 | 7.492 | 2.40-23.4 | 0.0005 | 5.563 | 1.84-16.8 | 0.0023 |
| AML risk: intermediate vs. low | 3.618 | 1.39-9.45 | 0.0086 | 4.680 | 1.59-13.7 | 0.0050 | 3.092 | 1.07-8.95 | 0.0374 |
| Gender: male vs. female | 1.071 | 0.55-2.07 | 0.8389 | 1.412 | 0.66-3.01 | 0.3722 | 1.038 | 0.49-2.18 | 0.9219 |
| No HCT vs. HCT | 2.191 | 1.09-4.41 | 0.0281 | 5.381 | 2.15-13.5 | 0.0003 | 2.094 | 0.96-4.58 | 0.0643 |
| Treatment group: SDC vs. LDC/G-CSF | 0.785 | 0.37-1.66 | 0.5276 | 0.604 | 0.26-1.41 | 0.2435 | 0.744 | 0.32-1.71 | 0.4849 |

Abbreviations: CI, confidence interval; EFS, event-free survival; HR, hazard ratio; LDC/G-CSF, low-dose chemotherapy concurrent with G-CSF; OS, overall survival; SDC, standard-dose chemotherapy; WBC, white blood cell; HCT, hematopoietic cell transplant.

### Supplementary Table 3. Cox Regression Analysis with Treatment, Gender, Age, Initial WBC, AML Risk, and HCT as Covariates in Patients with Initial WBC Count <70×10^9^/L.

| **Clinical Feature** | **EFS** | | | **OS** | | | **Any Relapse** | | |
| --- | --- | --- | --- | --- | --- | --- | --- | --- | --- |
|  | **HR** | **95% CI** | **P value** | **HR** | **95% CI** | **P value** | **HR** | **95% CI** | **P value** |
| Age: older than 10 vs. <10 years | 2.233 | 0.88-5.70 | 0.0926 | 2.506 | 0.90-7.00 | 0.0797 | 1.723 | 0.56-5.26 | 0.3392 |
| WBC group 50≤WBC<100 vs. <50 | 0.494 | 0.06-3.80 | 0.4977 | - | - | 0.9919 | 0.667 | 0.09-5.22 | 0.6995 |
| AML risk: high vs. low | 4.257 | 1.38-13.2 | 0.0119 | 4.020 | 1.08-15.0 | 0.0384 | 4.347 | 1.26-15.0 | 0.0199 |
| AML risk: intermediate vs. low | 3.042 | 1.09-8.51 | 0.0341 | 3.656 | 1.16-11.6 | 0.0273 | 2.685 | 0.84-8.54 | 0.0941 |
| Gender: male vs. female | 0.851 | 0.39-1.85 | 0.6850 | 1.236 | 0.51-2.99 | 0.6376 | 0.742 | 0.31-1.76 | 0.4983 |
| No HCT vs. HCT | 2.129 | 0.92-4.95 | 0.0792 | 8.809 | 2.01-38.6 | 0.0039 | 1.865 | 0.75-4.66 | 0.1820 |
| Treatment group: SDC vs. LDC/G-CSF | 0.753 | 0.35-1.60 | 0.4616 | 0.573 | 0.24-1.35 | 0.2014 | 0.718 | 0.31-1.65 | 0.4364 |

Abbreviations: AML, acute myeloid leukemia; HCT, hematopoietic cell transplant; CI, confidence interval; EFS, event-free survival; HR, hazard ratio; LDC/G-CSF, low-dose chemotherapy concurrent with G-CSF; OS, overall survival; SDC, standard-dose chemotherapy; WBC, white blood cell.

### Supplementary Table 4. Toxicity and Cost by Treatment Group.

| **Feature** | **LDC/G-CSF**  **N (%)** | **SDC**  **N (%)** | **Total**  **N (%)** | ***P*-value** |
| --- | --- | --- | --- | --- |
| **First Remission Induction** | 46 | 94 | 140 |  |
| Duration of neutropenia—Days |  |  |  | <0.001^a^ |
| Median | 11.5 | 18.5 | 17.0 |  |
| Range | 0.0–30.0 | 2.0–60.0 | 0.0–60.0 |  |
| Duration of thrombocytopenia—Days |  |  |  | <0.001^a^ |
| Median | 15.5 | 22.0 | 21.0 |  |
| Range | 0.0–36.0 | 6.0–45.0 | 0.0–45.0 |  |
| Hepatic toxicity—N (%) |  |  |  | 1.00 ^b^ |
| 0–II | 44 (95.7) | 90 (95.7) | 134 (95.7) |  |
| III–IV | 2 (4.3) | 4 (4.3) | 6 (4.3) |  |
| Infections—N (%) |  |  |  | <0.001^b^ |
| None | 16 (34.8) | 2 (2.1) | 18 (12.9) |  |
| I | 10 (21.7) | 10 (10.7) | 20 (14.3) |  |
| II | 16 (34.8) | 59 (62.8) | 75 (53.6) |  |
| III | 3 (6.5) | 21 (22.3) | 24 (17.1) |  |
| IV | 1 (2.2) | 2 (2.1) | 3 (2.1) |  |
| Cost of first induction—US Dollars |  |  |  | <0.001^a^ |
| Median | 7,463 | 10,315 | 9,474 |  |
| Range | 1,916–20,189 | 3,762–28,926 | 1,916–28,926 |  |
|  | | | | |
| **Second Remission Induction** | 28 | 90 | 118 |  |
| Duration of neutropenia—Days |  |  |  | <0.001^a^ |
| Median | 6.5 | 12.0 | 12.0 |  |
| Range | 0.0–16.0 | 4.0–36.0 | 0.0–36.0 |  |
| Duration of thrombocytopenia—Days |  |  |  | <0.001^a^ |
| Median | 11.5 | 17.0 | 15.0 |  |
| Range | 0.0–42.0 | 7.0–36.0 | 0.0–42.0 |  |
| Hepatic toxicity—N (%) |  |  |  | 1.00 ^b^ |
| 0–II | 27 (96.4) | 86 (95.6) | 113 (95.8) |  |
| III–IV | 1 (3.6) | 4 (4.4) | 5 (4.2) |  |
| Infections —N (%) |  |  |  | <0.001^b^ |
| None | 20 (71.4) | 0 (0.0) | 20 (16.9) |  |
| I | 5 (17.9) | 13 (14.4) | 18 (15.3) |  |
| II | 1 (3.6) | 63 (70.0) | 64 (54.2) |  |
| III | 2 (7.1) | 10 (1.1) | 12 (10.2) |  |
| IV | 0 (0.0) | 4 (4.5) | 4 (3.4) |  |
| Cost of second induction—U.S. Dollars |  |  |  | <0.001^a^ |
| Median | 2,363 | 5,719 | 4,992 |  |
| Range | 1,332–8,258 | 1,748–21,118 | 1,332–21,118 |  |

Abbreviations: LDC/G-CSF, low-dose chemotherapy concurrent with G-CSF; SDC, standard-dose chemotherapy

^a^Wilcoxon rank-sum test.

^b^Exact Pearson's chi-squared test.

### Supplementary Table 5. Clinical and Genomic Characteristics at Diagnosis for Patients Whose Samples were Analyzed by Ampliseq.

|  | | **Group** | |  |
| --- | --- | --- | --- | --- |
| **Parameters** | **Total**  **(n=20)** | **LDC/G-CSF**  **(n=9)** | **SDC**  **(n=11)** | ***P*-value** |
| **Age at diagnosis—months** |  |  |  | 0.569 ^a^ |
| Median | 97 | 89 | 109 |  |
| Range | 22-166 | 22-141 | 34-166 |  |
| **WBC** |  |  |  | 0.184 ^a^ |
| Median | 23.275 | 14.86 | 30.6 |  |
| Range | 3.12-205.49 | 3.12–41.78 | 4.7–205.49 |  |
| **HGB（×g/L）** |  |  |  | 0.493 ^a^ |
| median±SD（range） | 84±16.5(46.2-108) | 78±16.6 (62–108) | 88±17.1 (46.2–103) |  |
| **PLT（×10^9^/L）** |  |  |  | 0.517 ^a^ |
| median±SD（range） | 45±43.2(13-179) | 38±42.2 (13–138) | 50±46 (19–179) |  |
| **Gender—n (%)** |  |  |  | 0.423 ^b^ |
| Female | 6(30) | 4 (44.4) | 3(27.3) |  |
| Male | 14(70) | 5 (55.6) | 8(72.7) |  |
| **Molecule—n (%)** |  |  |  | 0.528 ^b^ |
| AML-ETO1 | 10(50) | 4(44.4) | 6 (54.5) |  |
| MLLs | 2(10) | 1(11.1) | 1 (9.1) |  |
| CBF/MYH11 | 4(20) | 1(11.1) | 3 (27.3) |  |
| Negative | 4(20) | 3(33.3) | 1 (9.1) |  |
| **Gene Mutation—n (%)** |  |  |  | 0.718 ^b^ |
| C-kit | 5(25) | 2(22.2) | 3 (27.3) |  |
| *NPM1* | 1(5) | 1(11.1) | 0 (0) |  |
| *FLT3*-ITD | 0(0) | 0(0) | 0 (0) |  |
| CEBPα double mutation | 2(10) | 1(9.1) | 1 (9.1) |  |
| Negative | 12(60) | 5(55.6) | 7 (63.6) |  |
| **Karyotype—n (%)** |  |  |  | 0.419 ^b^ |
| Favorable | 12(60) | 5(55.6) | 7(63.6) |  |
| Intermediate | 6(30) | 2(22.2) | 4(36.4) |  |
| Adverse | 1(5) | 1(11.1) | 0(0) |  |
| Undetectable*^1^ | 1(5) | 1(11.1) | 0(0) |  |
| **Coverage** |  |  |  |  |
| Median | 12362 | 13179.5 | 12345 | 0.6409^a^ |
| Range | 307-41318 | 307-27819 | 566-41318 |  |
| **Number of mutations**  **(at diagnosis)** |  |  |  | 0.267 ^a^ |
| Mean | 6.9 | 7.6 | 6.3 |  |
| Range | 2-13 | 4-13 | 2-13 |  |

Abbreviation: LDC/G-CSF, low-dose chemotherapy concurrent with G-CSF; SDC, standard-dose chemotherapy; HGB, hemoglobin; PLT, platelet count.

^a^Wilcoxon rank-sum test.

^b^Exact Pearson’s chi-squared test.

### Supplementary Table 6. Leukemia-Associated Recurrent Mutated Genes (n=268) Used in This Study.

| *ABCG2* | *CNTN5* | *FAM47A* | *ITPR3* | *MTA2* | *PRPF4B* | *SUZ12* |
| --- | --- | --- | --- | --- | --- | --- |
| *ABTB1* | *CNTNAP4* | *FAM57B* | *JAK1* | *MTMR8* | *PRPF8* | *SYNGAP1* |
| *ADAM11* | *COL12A1* | *FAM5C* | *JAK2* | *MTUS2* | *PSME4* | *SYT15* |
| *ADCY5* | *COL5A3* | *FAM65A* | *JAK3* | *MUC16* | *PTCH1* | *TCEAL3* |
| *AKAP13* | *CRISPLD1* | *FAM70B* | *KCNA4* | *MUC5B* | *PTPN11* | *TCEAL6* |
| *ALPK3* | *CROCC* | *FCGBP* | *KCNH2* | *MYC* | *PTPRN* | *TET1* |
| *ANK2* | *CSMD1* | *FKBP8* | *KCNK13* | *MYH4* | *PTPRT* | *TET2* |
| *APOB* | *CSMD3* | *FLG* | *KCNQ2* | *MYO5B* | *RAD21* | *THRAP3* |
| *ARAP2* | *CUEDC1* | *FLJ43860* | *KCNT1* | *MYOC* | *RBBP4* | *TMEM104* |
| *ASXL1* | *CUL1* | *FLRT2* | *KCNU1* | *MYOM3* | *RFC3* | *TNC* |
| *ASXL2* | *DCHS2* | *FLT1* | *KDM3B* | *NALCN* | *RIMS1* | *TOP3B* |
| *ATG16L1* | *DCLK1* | *FLT3* | *KDM6A* | *NAV1* | *RNF213* | *TP53* |
| *ATP10B* | *DDR2* | *FOXP1* | *KDR* | *NF1* | *RUNX1* | *TRA2B* |
| *ATP2B3* | *DDX11* | *FREM2* | *KIAA0240* | *NLRC4* | *RUNX1T1* | *TRPM3* |
| *BCOR* | *DDX41* | *FRYL* | *KIAA1267* | *NMUR2* | *RYR1* | *TTBK1* |
| *BCORL1* | *DHX30* | *GALNTL4* | *KIAA1529* | *NPM1* | *RYR3* | *TUBA3C* |
| *BMPER* | *DIS3* | *GAS6* | *KIAA1683* | *NR2E1* | *SBF1P1* | *TYK2* |
| *BOD1L* | *DLC1* | *GATA2* | *KIF2B* | *NRAS* | *SCAF8* | *U2AF1* |
| *BSN* | *DNAH5* | *GBP4* | *KIT* | *NRXN3* | *SCARB1* | *UNC5B* |
| *C10orf118* | *DNAH9* | *GIGYF2* | *KRAS* | *NTRK3* | *SCML2* | *USP9X* |
| *C10orf28* | *DNAI1* | *GJB3* | *KRT19* | *OR11H12* | *SCN1A* | *VARS2* |
| *C17orf97* | *DNMT3A* | *GPR112* | *KRT79* | *OR13H1* | *SEMA3A* | *VCAN* |
| *C5* | *DNMT3B* | *GPR183* | *KSR2* | *OR8B12* | *SEMA4A* | *WAC* |
| *CACNA1B* | *DOCK2* | *GRID1* | *LNX1* | *P2RY2* | *SETBP1* | *WT1* |
| *CACNA1E* | *DRD2* | *GRIK2* | *LOC100129218* | *PCDHA13* | *SF3B1* | *XIRP1* |
| *CACNA1G* | *DSCAM* | *GRIK4* | *LOC100132800* | *PCDHA6* | *SHC1* | *ZBTB33* |
| *CACNA2D3* | *DST* | *GRM3* | *LOC730032* | *PCDHB1* | *SHROOM2* | *ZC3H18* |
| *CADM2* | *DYNC2H1* | *GRM8* | *LRBA* | *PCDHB18* | *SI* | *ZNF687* |
| *CADPS* | *DYSF* | *GSTK1* | *LRIT1* | *PDCD2L* | *SLC12A3* |  |
| *CALR* | *E2F8* | *HECW1* | *LRP1B* | *PHACTR1* | *SLC39A5* |  |
| *CBFB* | *EDIL3* | *HIVEP1* | *LRRC4* | *PHF6* | *SMC1A* |  |
| *CBL* | *EED* | *HMCN1* | *MAGI2* | *PHIP* | *SMC3* |  |
| *CCDC67* | *EEF1A1P29* | *HNRNPK* | *MAP1B* | *PKD1L2* | *SMG1* |  |
| *CD74* | *EGFR* | *HSP90B3P* | *MAP2* | *PKD2L1* | *SPEG* |  |
| *CEBPA* | *EPHA10* | *HYDIN* | *MED12* | *PKHD1* | *SPEN* |  |
| *CECR2* | *EPPK1* | *IDH1* | *MEFV* | *PKHD1L1* | *SRSF2* |  |
| *CELSR3* | *ETV6* | *IDH2* | *MEGF8* | *PLCE1* | *ST13P13* |  |
| *CHD4* | *EZH2* | *IGHG3* | *MIR142* | *PLEKHH1* | *STAG2* |  |
| *CLEC18B* | *FAM154B* | *IKZF4* | *MLL3* | *PPP1R3A* | *STC2* |  |
| *CMYA5* | *FAM40B* | *ILDR1* | *MPL* | *PRAMEF2* | *STRN* |  |
|  |  |  |  |  |  |  |

### Supplementary Table 7. Children's Hospital of Soochow University Pediatric Acute Myeloid Leukemia Hematopoietic Cell Transplant Regimens.

|  | **Drug** | **Dose** | **Schedule** | **Days** |
| --- | --- | --- | --- | --- |
| **Conditioning** |  |  |  |  |
| Matched sibling | Cytarabine | 2 g/m^2^ | Every 12 h | –8, –7 |
|  | Busulfan | 0.8 mg/kg | Every 6 h | –6, –5, –4 |
|  | Cyclophosphamide | 60 mg/kg | Once daily | –3, –2 |
|  | Semustine | 250 mg/m^2^ | Once daily, oral | –9 |
|  | Hydroxyurea | 40mg/kg | Every 12 h | –9 |
| Haploidentical | Cytarabine | 2 g/m^2^ | Every 12 h | –8, –7 |
|  | Busulfan | 0.8 mg/kg | Every 6 h | –6, –5, –4 |
|  | Cyclophosphamide | 1.8 g/m^2^ | Once daily | –3, –2 |
|  | Semustine | 250 mg/m^2^ | Once daily, oral | –9 |
|  | ATG | 2.5 mg/kg | Once daily | –5, –4, –3, –2 |
| Unrelated donor | Cytarabine | 2 g/m^2^ | Every 12 h | –10, –9 |
|  | Busulfan | 0.8 mg/kg | Every 6 h | –8, –7, –6 |
|  | Cyclophosphamide | 1.8 g/m^2^ | Once daily | –5, –4 |
|  | Semustine | 250 mg/m^2^ | Once daily, oral | –3 |
|  | ATG | 2.5 mg/kg | Once daily | –5, –4 |
| Cord blood | Cytarabine | 2 g/m^2^ | Every 12 h | –5, –4 |
|  | Busulfan | 0.8 mg/kg | Every 6 h | –7, –6, –5, –4 |
|  | Cyclophosphamide | 60 mg/kg | Once a day | ­–3, –2 |
| Graft vs. host prophylaxis | Methotrexate | 15 mg/m^2^  10 mg/m^2^ | Once daily | +1  +3, +6, +11 |
|  | Cyclosporin A | 1.5 mg/kg | Every 12 h | Since –3 |
|  | Mycophenolate Mofetil | 15–20 mg/kg | Every 12 h | +1 to +30 |

Abbreviations: ATG, anti-thymocyte globulin; GVHD, graft-versus-host disease.

### Supplementary Table 8. Cox Regression Analysis with Treatment, Gender, Age, Initial WBC Count, Final Risk, and HSC as Covariates.

| **Clinical Feature** | **EFS** | | | **OS** | | | **Any Relapse** | | |
| --- | --- | --- | --- | --- | --- | --- | --- | --- | --- |
|  | **HR** | **95% CI** | **P value** | **HR** | **95% CI** | **P value** | **HR** | **95% CI** | **P value** |
| Age: 1–10 years vs. infant | 1.565 | 0.36-6.86 | 0.5527 | 1.103 | 0.25-4.96 | 0.8979 | 2.694 | 0.35-20.7 | 0.3406 |
| Age: Older than 10 years vs. infant | 2.672 | 0.54-13.1 | 0.2261 | 2.096 | 0.42-10.5 | 0.3694 | 4.147 | 0.48-35.8 | 0.1956 |
| WBC group: 50≤WBC<100 vs. <50 | 0.530 | 0.12-2.29 | 0.3944 | 0.410 | 0.05-3.12 | 0.3898 | 0.676 | 0.15-2.99 | 0.6057 |
| WBC groups WBC≥100 vs. <50 | 1.049 | 0.42-2.64 | 0.9184 | 1.373 | 0.49-3.84 | 0.5452 | 0.807 | 0.28-2.29 | 0.6867 |
| Final risk: High/very high vs. low | 5.153 | 1.65-16.1 | 0.0048 | 5.512 | 1.49-20.4 | 0.0105 | 6.259 | 1.74-22.6 | 0.0051 |
| Final risk: Intermediate vs. low | 2.041 | 0.66-6.35 | 0.2184 | 2.470 | 0.67-9.11 | 0.1746 | 1.846 | 0.49-6.94 | 0.3646 |
| Gender: male vs. female | 0.858 | 0.45-1.65 | 0.6458 | 1.043 | 0.49-2.21 | 0.9118 | 0.873 | 0.42-1.82 | 0.7166 |
| No HCT vs. HCT | 1.717 | 0.88-3.34 | 0.1107 | 3.620 | 1.54-8.51 | 0.0032 | 1.658 | 0.78-3.51 | 0.1865 |
| Treatment group: SDC vs. LDC/G-CSF | 0.821 | 0.39-1.72 | 0.6025 | 0.623 | 0.27-1.44 | 0.2687 | 0.783 | 0.34-1.78 | 0.5600 |

Abbreviations: HCT, hematopoietic cell transplant. CI, confidence interval; EFS, event-free survival; HR, hazard ratio; LDC/G-CSF, low-dose chemotherapy concurrent with G-CSF; OS, overall survival; SDC, standard-dose chemotherapy; WBC, white blood cell.

### Supplementary Table 9. Cox Regression Analysis with Treatment, Gender, Age, Initial WBC, Final Risk, and HCT as Covariates in Patients with initial WBC<70×10^9^/L.

| **Clinical Feature** | **EFS** | | | **OS** | | | **Any Relapse** | | |
| --- | --- | --- | --- | --- | --- | --- | --- | --- | --- |
|  | **HR** | **95% CI** | **P value** | **HR** | **95% CI** | **P value** | **HR** | **95% CI** | **P value** |
| Age: Older than 10 vs. <10 years | 1.974 | 0.79-4.90 | 0.1432 | 2.297 | 0.83-6.33 | 0.1079 | 1.520 | 0.51-4.51 | 0.4512 |
| WBC groups: 50≤WBC<100 vs. <50 | 0.557 | 0.07-4.32 | 0.5761 | 0.000 | 0.00- | 0.9924 | 0.791 | 0.10-6.32 | 0.8248 |
| Final risk: High/very high vs. low | 4.802 | 1.53-15.0 | 0.0071 | 5.429 | 1.46-20.2 | 0.0115 | 5.463 | 1.51-19.8 | 0.0096 |
| Final risk: intermediate vs. low | 2.141 | 0.68-6.71 | 0.1916 | 3.000 | 0.81-11.1 | 0.1004 | 1.869 | 0.49-7.08 | 0.3572 |
| Gender: male vs. female | 0.790 | 0.37-1.69 | 0.5431 | 1.115 | 0.47-2.63 | 0.8032 | 0.721 | 0.31-1.68 | 0.4475 |
| No HCT vs. HCT | 2.121 | 0.92-4.91 | 0.0792 | 8.709 | 1.99-38.1 | 0.0041 | 1.820 | 0.73-4.54 | 0.1989 |
| Treatment groups: SDC vs. LDC/G-CSF | 0.763 | 0.36-1.61 | 0.4792 | 0.574 | 0.24-1.35 | 0.2025 | 0.723 | 0.32-1.66 | 0.4433 |

Abbreviations: BMT, bone marrow transplant; CI, confidence interval; EFS, event-free survival; HR, hazard ratio; LDC/G-CSF, low-dose chemotherapy concurrent with G-CSF; OS, overall survival; SDC, standard-dose chemotherapy; WBC, white blood cell.

# Supplementary References

1. Beillard E, Pallisgaard N, van der Velden VH, et al. Evaluation of candidate control genes for diagnosis and residual disease detection in leukemic patients using 'real-time' quantitative reverse-transcriptase polymerase chain reaction (RQ-PCR) - a Europe against cancer program. *Leukemia.* 2003;17(12):2474-2486.
